# Time‐correlated model error in the (ensemble) Kalman smoother

**DOI:** 10.1002/qj.3378

**Published:** 2018-10-28

**Authors:** Javier Amezcua, Peter Jan van Leeuwen

**Affiliations:** ^1^ Department of Meteorology and National Centre for Earth Observation, University of Reading, UK

**Keywords:** data assimilation, ensembles, model error, statistical methods

## Abstract

Data assimilation is often performed in a perfect‐model scenario, where only errors in initial conditions and observations are considered. Errors in model equations are increasingly being included, but typically using rather adhoc approximations with limited understanding of how these approximations affect the solution and how these approximations interfere with approximations inherent in finite‐size ensembles.

We provide the first systematic evaluation of the influence of approximations to model errors within a time window of weak‐constraint ensemble smoothers. In particular, we study the effects of prescribing temporal correlations in the model errors incorrectly in a Kalman smoother, and in interaction with finite‐ensemble‐size effects in an ensemble Kalman smoother.

For the Kalman smoother we find that an incorrect correlation time‐scale for additive model errors can have substantial negative effects on the solutions, and we find that overestimating of the correlation time‐scale leads to worse results than underestimating. In the ensemble Kalman smoother case, the resulting ensemble‐based space–time gain can be written as the true gain multiplied by two factors, a linear factor containing the errors due to both time‐correlation errors and finite ensemble effects, and a nonlinear factor related to the inverse part of the gain. Assuming that both errors are relatively small, we are able to disentangle the contributions from the different approximations. The analysis mean is affected by the time‐correlation errors, but also substantially by finite‐ensemble effects, which was unexpected. The analysis covariance is affected by both time‐correlation errors and an in‐breeding term. This first thorough analysis of the influence of time‐correlation errors and finite‐ensemble‐size errors on weak‐constraint ensemble smoothers will aid further development of these methods and help to make them robust for e.g. numerical weather prediction.

## 1 INTRODUCTION

Data assimilation (DA) combines incomplete and imperfect sources of information of a system to obtain a better estimate of that system, including uncertainties. These sources – models and observations for example – can be represented as random variables with given probability density functions (pdfs). The reader is referred to e.g. van Leeuwen *et al.* (2015) and Ash *et al.* (2017), for recent introductions.

The general solution to the DA problem is given by Bayes' theorem (Bayes and Price, 1763):

(1) $$p(x|y)=\frac{p(y|x)p(x)}{p(y)}$$

In Equation 1 the *background* information of the *state variables* is contained in the prior pdf *p*(**x**). Observations become available over time and their information – on at least a subset of the state variables – is contained in the likelihood *p*(**y**|**x**). The posterior pdf *p*(**x**|**y**) – the probability of the state variables given the observations – is the result of multiplying prior and likelihood. The denominator *p*(**y**) is the marginal pdf of the observations and does not depend on the state variables. Hence, DA can be regarded as a multiplication problem involving different sources of information.

We seldom work with pdfs in practice because, as soon as the dimension of the system is larger than (say) 3, we have difficulty storing and propagating full pdfs. Most DA methods rely on estimating statistics of the posterior pdf and are based – to different degrees – on assumptions of Gaussianity in the error sources. Most methods are optimal when the evolution of the system and the observation process are linear. Variational methods like 4D‐Var (Le Dimet and Talagrand, 1986; Talagrand and Courtier, 1987) work with the mode of the posterior pdf, whereas methods based on the Kalman filter (KF; Kalman, 1960; Kalman and Bucy, 1961) and its ensemble implementations (e.g. the EnKF; Evensen, 1994; Burgers *et al.*, 1998) work with the first two moments.

DA often works with systems that evolve in time. This time dependence can be handled in two ways. Filters update the state variables only at the observed times, whereas smoothers update whole trajectories of the state variables in a given assimilation window, using simultaneously all the observations available during that time frame. In this work we focus on the latter.

Consider that the true evolution of the system is generated by a *true model*, and that the forecast step of the DA process is generated by a *forecast model*. If these two models perfectly match, we say we work in a strong‐constraint (SC) framework. This situation reduces the smoothing problem to searching for the initial conditions of the assimilation window. In geosciences, however, there is always a mismatch between the true model and the forecast model. This mismatch is called *model error*, and it can arise from the discretization of the underlying partial differential equations describing the system, the parametrization of processes that cannot be explicitly resolved, the lack of knowledge of some physical processes, and many other sources. The reader is referred to Howles *et al.* (2017) for a further discussion. Sometimes model error is small enough to be ignored compared to other uncertainties, but this is not always the case.

Model error can be simulated in different forms within the DA forecast step. It can be inserted as a random additive term at every given number of model time steps, or as a random multiplicative factor in the tendencies of the model equations (Palmer *et al.*, 2009). More indirect ways include using different parametrization schemes for different ensemble members, which means we can sometimes represent model error even without clearly knowing its statistics. Including any existing model error is particularly important in ensemble forecasting, since it is needed to produce a good estimate of the actual forecast uncertainty.

Even when it is present in the DA forecast step, model error is often not treated explicitly in the DA analysis step (e.g. Bonavita *et al.*, 2016). Treating model error in the DA analysis step is known as *weak‐constraint* (WC) framework; the reader is referred to Sasaki (1970) and Tremolet (2006) for an introduction. A WC smoother poses a considerably harder problem for several reasons. Firstly, model error statistics are often unknown, and although some methods have arisen to estimate them (e.g. Todling, 2015; Zhu *et al.*, 2018), this is a challenging task. Secondly, the size of the control variable and the computational expense of the problem is larger than in the SC case. This is because not only the initial conditions but intermediate jumps need to be estimated as well. To reduce this burden one can consider “effective” model errors over a large number of time steps (Tremolet, 2006).

Since specifying the correct model error statistics is not trivial, it is important to understand the consequences of using an incorrect time‐autocorrelation of the model error in a WC smoother. This is a question we aim to answer in this paper. Under linear model evolution and linear observation operators, the problem we study is equivalent to the inner loops of an ensemble of WC 4D‐Vars with perturbed predicted observations and additive model errors for each trajectory. Since the (full) nonlinear problem is often solved as a sequence of successively linearized problems, our results are also relevant for the nonlinear case.

For many dynamical systems of interest, the background‐error covariance used in the prior *p*(**x**) evolves with time. One way to find a time‐evolving estimator of this covariance is to use an ensemble, as in the case of ensemble Kalman filters; Vetra‐Carvalho *et al.* (2017) give a review on the implementation of several different flavours. Furthermore, the use of hybrid ensemble‐variational methods has grown in the last years, and these are seen by many as a pathway to the future for e.g. numerical weather prediction. The reader is referred to Goodliff *et al.* (2015) and the references therein for an overview.

As discussed, sample information is important in many DA methods, but the finite‐sized nature of an ensemble method introduces error. Besides the direct sampling (Monte‐Carlo) error, there is a more subtle indirect sampling error which comes from the use of sample covariance statistics in the gain required in the analysis step of the smoothers. These issues were recognised by Houtekamer and Mitchel (1998) and analysed by van Leeuwen (1999), and studied in more detail by Sacher and Bartello (2008) and Furrer and Bengsston (2007) in the filtering setting. In this paper we identify and quantify the effects of both direct and indirect sampling errors in the ensemble Kalman smoother. Furthermore, we study the interactions between these finite‐size sample effects and the errors arising from incorrect specification of the temporal‐correlation, or memory, of the model error. This is done within a single (forecast/assimilation) time window. Future work will explore the effect of cycling, and the extension into nonlinear systems.

The paper is structured as follows. In section 2 we derive the exact analysis solution for a time window of the ensemble smoother in the presence of autocorrelated additive Gaussian model error. For an exponential autocorrelation memory function, we illustrate the propagation of information from observations inside the assimilation window. This exact solution serves as benchmark for the work in section 3 where we introduce two sources of imperfection: a mis‐specification of the autocorrelation memory of the model error, as well as the use of finite‐size ensembles. Section 4 illustrates the difference between direct and indirect sampling errors arising from an ensemble using numerical examples. Section 5 provides a summary and conclusions. This work is heavy in equations. To aid the reader we have underlined the most important expressions which often have significance throughout the whole work.

## 2 KALMAN SMOOTHER WITH TEMPORAL‐CORRELATED MODEL ERROR

Let us consider a system akin to that of Howles *et al.* (2017), although our ultimate purpose is different. We denote the state variable at *t* = 0 as $x^{0}\in R^{N_{x}}$. This random variable **x** ^0^ follows a multivariate Gaussian distribution (MGD) $x^{0}∼N\left(\mu_{x}^{0,b},B\right)$, where $\mu_{x}^{0,b}\in R^{N_{x}}$ is its mean, $B\in R^{N_{x}\times N_{x}}$ is its covariance matrix, and the superscript b stands for background. Over one time step the state variable evolves as

(2) $$x^{t}=M^{(t-1)\to t}x^{t-1}+v^{t}.$$

The linear operator $M^{(t-1)\to t}\in R^{N_{x}\times N_{x}}$ has the property

(3) $$M^{t_{1}\to t_{3}}=M^{t_{2}\to t_{3}}M^{t_{1}\to t_{2}}$$

for 0 ≤ *t*_1_ < *t*_2_ < *t*_3_. The variable $v^{t}\in R^{N_{x}}$ is the model error jump at time step *t*. This random variable has a MGD $v^{t}∼N\left(0,Q\right)$, with mean $0\in R^{N_{x}}$ and model error covariance matrix $Q\in R^{N_{x}\times N_{x}}$. The model error jumps can be correlated in time:

(4) $$Cov(v^{i},v^{j})=\phi \left(|i-j|,\omega \right)Q,$$

where 0 ≤ *ϕ* ≤ 1 represents the memory, |*i* − *j*| is the absolute difference between time steps *i* and *j*, and *ω* is a characteristic memory time‐scale of the system. The function *ϕ* takes the value 1 when |*i* − *j*| = 0, and decreases monotonically towards 0 as |*i* − *j*| increases. We keep our work general for any function *ϕ* that fulfils these two conditions, but for more specific examples we use an exponentially decaying memory:

(5) $$\phi \left(|i-j|,\omega \right)=e^{-\frac{\left|i-j\right|}{\omega }}.$$

Observations are taken every Δ_*obs*_ time steps. The *l*th observation $y^{l}\in R^{N_{y}}$ is obtained as:

(6) $$y^{l}=H^{l}x^{t}+\eta^{l},$$

where $H^{l}\in R^{N_{y}\times N_{x}}$ is the *l*th linear observation operator, and $\eta^{l}\in R^{N_{y}}$ is the observational error. This random variable follows a zero‐mean MGD $\eta^{l}∼N\left(0,R\right)$, where $R\in R^{N_{y}\times N_{y}}$ is the observational‐error covariance. The random variables **x** ^0^, ***η*** and **v** ^*t*^ are assumed to be statistically independent of each other.

For brevity in the derivations, we consider the time window *t* = {0,1,⋯,*τ* − 1,*τ*} to contain only one observation at *t* = *τ*, i.e. at the end of the time window. This can be generalized to *L* observation in two ways. The first is to realise that – in the case of linear model and observation operators – observations at different times can be assimilated sequentially. This is akin to the serial EnKF of Whitaker and Hamill (2002). In the case of assimilating all observational times at once, the following derivations are still valid when using the extended expressions of the Appendix.

The collection of model error jumps can be written as one long vector $v^{1:\tau }\in R^{\tau N_{x}}$:

(7) $$v^{1:\tau }={\left[{\left(v^{1}\right)}^{T},{\left(v^{2}\right)}^{T},\text{⋯}\;,{\left(v^{\tau }\right)}^{T}\right]}^{T}.$$

This random variable follows a MGD $v^{1:\tau }\;∼\;N\left(0,Q^{1:\tau }\right)$, with mean $0\in R^{\tau N_{x}}$ and covariance $Q^{1:\tau }\in R^{\tau N_{x}\times \tau N_{x}}$, which is a block‐matrix in which each element contains **Q** multiplied by a memory coefficient. It is helpful to write **Q** ^1:*τ*^ as the Kronecker (outer) product:

(8) $$Q^{1:\tau }=\Phi^{1:\tau }⊗Q,$$

where $\Phi^{1:\tau }\in R^{\tau ,\tau }$ is a Toeplitz matrix of memory coefficients:

(9) $$\Phi^{1:\tau }=\left[\begin{array}{ccccc} 1 & \phi (1,\omega ) & \text{⋯} & \phi (\tau -2,\omega ) & \phi (\tau -1,\omega )\\ \phi (1,\omega ) & 1 & \text{⋯} & \phi (\tau -3,\omega ) & \phi (\tau -2,\omega )\\ \vdots & \vdots & \text{⋯} & \vdots & \vdots \\ \phi (\tau -2,\omega ) & \phi (\tau -3,\omega ) & \text{⋯} & 1 & \phi (1,\omega )\\ \phi (\tau -1,\omega ) & \phi (\tau -2,\omega ) & \text{⋯} & \phi (1,\omega ) & 1 \end{array}\right]$$

**Figure 1:**
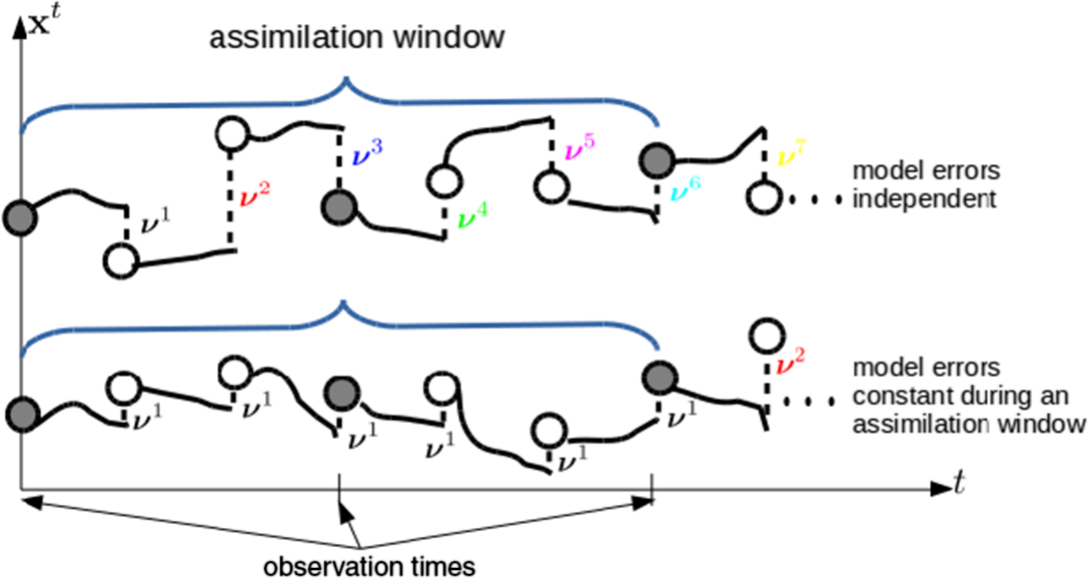
Schematic illustration of the evolution of a state variable throughout an assimilation window, showing the initial conditions at t = 0, deterministic evolution (solid lines), and model error jumps (dashed lines). The top row shows the case of independent model errors (zero temporal correlation), while the bottom row shows the case of fixed model errors (perfect temporal correlation) [Colour figure can be viewed at wileyonlinelibrary.com]

It is useful to note two limiting cases: (i) The zero‐memory case occurs when *ω*→0, yielding *ϕ*(|*i* − *j*|,*ω*) = 0∀|*i* − *j*| > 0. Then **Φ** ^1:*τ*^ becomes the identity matrix, and **Q** ^1:*τ*^ becomes a block‐diagonal with **Q** in each diagonal block‐element. This corresponds to completely independent model error jumps. This is shown schematically in the top row of Figure 1. (ii) The infinite‐memory case occurs when *ω*→*∞*, yielding *ϕ*(|*i* − *j*|,*ω*) = 1∀|*i* − *j*|. Then **Φ** ^1:*τ*^ becomes a matrix of ones, and **Q** ^1:*τ*^ becomes a block‐matrix with **Q** in every block‐element. This corresponds to fixed model error jumps. This is shown schematically in the bottom row of Figure 1.

### 2.1 The weak‐constraint Kalman smoother

Now we describe the process performed by the WC Kalman smoother over one time window. Consider the extended control variable $z^{0:\tau }\in R^{(1+\tau )N_{x}}$ containing initial conditions and model error jumps:

(10) $$z^{0:\tau }={\left[{\left(x^{0}\right)}^{T},{\left(v^{1:\tau }\right)}^{T}\right]}^{T}$$

The posterior pdf *p*(**z** ^0:*τ*^|**y**) is simply:

(11) $$p(z^{0:\tau }|y)=\frac{p(y|z^{0:\tau })p(z^{0:\tau })}{p(y)},$$

where the prior *p*(**z** ^0:*τ*^) is a MGD $z^{0:\tau }∼N\left(\mu_{z}^{0:\tau ,b},D^{0:\tau }\right)$ with mean $\mu_{z}^{0:\tau ,b}\in R^{(1+\tau )N_{x}}$:

(12) $$\mu_{z}^{0:\tau ,b}=\left[\begin{array}{c} \mu_{x}^{0,b}\\ \mu_{v}^{1:\tau ,b} \end{array}\right]=\left[\begin{array}{c} \mu_{x}^{0,b}\\ 0 \end{array}\right]$$

and covariance matrix $D^{0:\tau }\in R^{(1+\tau )N_{x}\times (1+\tau )N_{x}}$. This matrix can be written in blocks as:

(13) $$D^{0:\tau }=\left[\begin{array}{cc} B & 0\\ 0 & Q^{1:\tau } \end{array}\right].$$

For later reference we note that we can formulate the problem in terms of the state variables **x** ^*τ*^ too via:

(14) $$x^{\tau }=M^{0:\tau }z^{0:\tau }$$

where $M^{0:\tau }\in R^{(1+\tau )N_{x}\times N_{x}}$ is the block‐matrix

(15) $$M^{0:\tau }=\left[M^{0\to \tau },M^{1\to \tau },M^{2\to \tau },\text{⋯}\;,M^{(\tau -1)\to \tau },I\right]$$

This equation exploits the linearity of the model, showing that each model error jump propagates independently of the rest of the variables to the end of the assimilation window where the observation is located. It allows us to write the likelihood *p*(**y**|**x** ^0:*τ*^) in terms of **z** ^0:*τ*^) as $p\left(y|z^{0:\tau }\right)$. Note that **y** is given, so the likelihood as function of **HM** ^0:*τ*^ **z** ^0:*τ*^ is given by $N\left(y,R\right)$. It is useful to compute the first two moments of **x** ^*τ*,*b*^ as:

(16) $$\begin{array}{ccc} E[x^{\tau ,b}] & =M^{0:\tau }\mu_{z}^{0:\tau ,b} & \\ Var[x^{\tau ,b}] & =M^{0:\tau }D^{0:\tau }{\left(M^{0:\tau }\right)}^{T} \end{array}$$

Performing the product in Equation 11 and doing some factorizations allows us to write the posterior as:

(17) $$p(z^{0:\tau }|y)\propto \exp\left[-\frac{1}{2}{\left(z^{0:\tau }-\mu_{z}^{0:\tau ,a}\right)}^{T}{\left(A_{z}^{0:\tau }\right)}^{-1}(z^{0:\tau ,a}-\mu_{z}^{0:\tau ,a})\right].$$

Therefore, the posterior pdf is:

(18) $$\underline{z^{0:\tau }|y∼N\left(\mu_{z}^{0:\tau ,a},A_{z}^{0:\tau }\right)},$$

with $\mu_{z}^{0:\tau ,a}\in R^{(1+\tau )N_{x}}$ defined as

(19) $$\mu_{z}^{0:\tau ,a}=\left(I-K_{z}^{0:\tau }HM^{0:\tau }\right)\mu_{z}^{0:\tau ,b}+K_{z}^{0:\tau }y$$

and $A_{z}^{0:\tau }\in R^{(1+\tau )N_{x}\times (1+\tau )N_{x}}$ defined as

(20) $$A_{z}^{0:\tau }=\left(I-K_{z}^{0:\tau }HM^{0:\tau }\right)D^{0:\tau }.$$

$K_{z}^{0:\tau }\in R^{(1+\tau )N_{x}\times N_{y}}$ is the Kalman gain in the extended‐variable space:

(21) $$K_{z}^{0:\tau }=D^{0:\tau }{\left(M^{0:\tau }\right)}^{T}H^{T}{\left(\Gamma^{\tau }\right)}^{-1},$$

where $\Gamma^{\tau }\in R^{N_{y}\times N_{y}}$ is the total covariance in observation space at the end of the assimilation window:

(22) $$\Gamma^{\tau }=HM^{0:\tau }D^{0:\tau }{\left(M^{0:\tau }\right)}^{T}H^{T}+R.$$

Finally, we note that in this Gaussian case $\mu_{z}^{0:\tau ,a}$ is also the minimizer of the cost function of the problem:

(23) $$\begin{array}{ccc} J(z^{0:\tau })= & \frac{1}{2}{\left(z^{0:\tau }-z^{0:\tau ,b}\right)}^{T}{\left(D^{0:\tau }\right)}^{-1}\left(z^{0:\tau }-z^{0:\tau ,b}\right) & \\ & +\frac{1}{2}{\left(y-HM^{0:\tau }z^{0:\tau }\right)}^{T}R^{-1}\left(y-HM^{0:\tau }z^{0:\tau }\right), \end{array}$$

which is nothing else but the minus logarithm of the numerator of Equation 11. This solution is standard knowledge (e.g. Jazwinski, 1970; Howles *et al.*, 2017). What follows, however, is our contribution. We examine in detail the effect that temporal correlation of model error has on the analysis values over the whole time window. We will study the solution in both initial condition‐model jump space and in state‐trajectory space.

### 2.2 Solution in terms of initial conditions and model error jumps

We now separate the components of the solution into those related to **x** ^0^ and those related to **v** ^1:*τ*^. We start by expanding Equation 22 as:

(24) $$\Gamma^{\tau }=HB^{\tau }H^{T}+H\Lambda^{\tau }H^{T}+R,$$

with $B^{\tau }\in R^{N_{x}\times N_{x}}$ and $\Lambda^{\tau }\in R^{N_{x}\times N_{x}}$ defined as:

(25) $$\begin{array}{ccc} B^{\tau } & =M^{0\to \tau }B{\left(M^{0\to \tau }\right)}^{T}, & \\ \Lambda^{\tau } & =M^{1:\tau }Q^{1:\tau }{\left(M^{1:\tau }\right)}^{T}. \end{array}$$

$B^{\tau }\in R^{N_{x}\times N_{x}}$ results from the evolution of **B**, and $\Lambda^{\tau }\in R^{N_{x},N_{x}}$ is the effective contribution of the model error. Explicitly, **Λ** ^*τ*^ is a double sum:

(26) $$\Lambda^{\tau }=\sum_{i=1}^{\tau }\sum_{j=1}^{\tau }M^{i\to \tau }Q{\left(M^{j\to \tau }\right)}^{T}\phi \left(|i-j|,\omega \right).$$

Using the short‐hand notation ${\tilde{M}}^{\tau }=\sum_{j=1}^{\tau }M^{j\to \tau }$ we can write the two limits:

(27) $$\lim_{\omega \to 0}\Lambda^{\tau }=\sum_{j=1}^{\tau }M^{j\to \tau }Q{\left(M^{j\to \tau }\right)}^{T},\lim_{\omega \to \infty }\Lambda^{\tau }={\tilde{M}}^{\tau }Q{\left({\tilde{M}}^{\tau }\right)}^{T}.$$

These limits show that when *ω*, and hence the memory, increases, the contribution of model error to the total covariance contains more terms and is expected to be larger. This, of course, is not surprising as a larger memory means that each random jump is felt long into the future.

The gain can be written as $K_{z}^{0:\tau }={\left[{\left(K_{x}^{0}\right)}^{T},{\left(K_{v}^{1:\tau }\right)}^{T}\right]}^{T}$, where the first component

(28) $$K_{x}^{0}=B{\left(M^{0\to \tau }\right)}^{T}H^{T}{\left(\Gamma^{\tau }\right)}^{-1}$$

acts on **x** ^0^ and the second

(29) $$K_{v}^{1:\tau }=Q^{1:\tau }{\left(M^{1:\tau }\right)}^{T}H^{T}{\left(\Gamma^{\tau }\right)}^{-1}$$

on **v** ^1:*τ*^. $K_{v}^{1:\tau }$ is a block‐matrix $K_{v}^{1:\tau }=[{\left(K_{v}^{1}\right)}^{T}\;{\left(K_{v}^{2}\right)}^{T}\text{⋯}\;$ ${\left(K_{v}^{\tau }\right)}^{T}]{}^{T}$ with the *j*th block‐element $K_{v}^{j}\in R^{N_{x}\times N_{y}}$ being

(30) $$K_{v}^{j}=Q\left(\sum_{i=1}^{\tau }{\left(M^{i\to \tau }\right)}^{T}\phi (|i-j|,\omega )\right)H^{T}{\left(\Gamma^{\tau }\right)}^{-1}.$$

Once more, the two limits of interest are:

(31) $$\lim_{\omega \to 0}K_{v}^{j}=Q{\left(M^{j\to \tau }\right)}^{T}H^{T}{\left(\Gamma^{\tau }\right)}^{-1},\lim_{\omega \to \infty }K_{v}^{j}={\left({\tilde{M}}^{\tau }\right)}^{T}H^{T}{\left(\Gamma^{\tau }\right)}^{-1}.$$

In the zero‐memory case, only one term in the sum (30) remains and it is different at every time step. In the infinite‐memory case, all terms in Equation 30 have the same coefficient *ϕ* = 1, and $K_{v}^{j}$ is exactly the same for all time steps.

With these expressions for the gains, we can formulate the full solution to the problem. We define $d\in R^{N_{y}}$ as departures of observations from evolved background:

(32) $$d=y-HM^{0\to \tau }\mu^{0,b}.$$

The analysis mean can then be written:

(33) $$\mu_{z}^{0:\tau ,a}=\left[\begin{array}{c} \mu_{x}^{0,a}\\ \mu_{v}^{1,a}\\ \vdots \\ \mu_{v}^{\tau ,a} \end{array}\right]=\left[\begin{array}{c} \mu_{x}^{0,b}+K_{x}^{0}d\\ K_{v}^{1}d\\ \vdots \\ K_{v}^{\tau }d \end{array}\right],$$

and the analysis covariance becomes:

(34) $$\begin{array}{ll} & A_{z}^{0:\tau }\;\\ & =\left[\begin{array}{cccc} (I-K_{x}^{0}HM^{0\to \tau })B & -K_{x}^{0}HM^{1\to \tau }Q & \text{⋯} & -K_{x}^{0}HQ\\ -K_{v}^{1}HM^{0\to \tau }B & (I-K_{v}^{1}HM^{1\to \tau })Q & \text{⋯} & -K_{v}^{1}HQ\\ \vdots & \vdots & \ddots & \vdots \\ -K_{v}^{\tau }HM^{0\to \tau }B & -K_{v}^{\tau }HM^{1\to \tau }Q & \text{⋯} & (I-K_{v}^{\tau }H)Q \end{array}\right].\; \end{array}$$

### 2.3 Solution in terms of state variables

We now express the solution in terms of the state variables at different times:

(35) $$x^{0:\tau ,a}={\left[{\left(x^{0,a}\right)}^{T}\;{\left(x^{1,a}\right)}^{T}\;\text{⋯}\;{\left(x^{\tau ,a}\right)}^{T}\right]}^{T}.$$

We can compute **x** ^*t*,*a*^ = **M** ^0:*t*^ **z** ^0:*t*,*a*^ as

(36) $$\underline{x^{t,a}=(I-K_{x}^{t}H)x^{t,b}+K_{x}^{t}y},$$

where **x** ^*t*,*b*^ = **M** ^0→*t*^ **x** ^0,*b*^. We introduce the new gain $K_{x}^{t}$ which acts directly on **x** ^*t*^ as

(37) $$K_{x}^{t}=M^{0\to t}K_{x}^{0}+\sum_{j=1}^{t}M^{j\to t}K_{v}^{j}.$$

The second term actually contains a double sum:

(38) $$\sum_{j=1}^{t}M^{j\to t}K_{v}^{j}=\left(\sum_{j=1}^{t}\sum_{i=1}^{\tau }M^{j\to t}Q{\left(M^{i\to \tau }\right)}^{T}\right)H^{T}{\left(\Gamma^{\tau }\right)}^{-1}.$$

Finally we compute the first two moments of **x** ^*t*,*a*^. The mean is

(39) $$\mu_{x}^{t,a}=(I-K_{x}^{t}H)\mu_{x}^{t,a}+K_{x}^{t}y$$

and the covariance

(40) $$A_{x}^{t}=\left(I-K_{x}^{t}H\right)\left(B^{t}+\Lambda^{t}\right),$$

where **B** ^*t*^ and **Λ** ^*t*^ are defined as in Equation 25 but for a general *t*.

### 2.4 Illustration in the scalar case

The effect of the temporally correlated model errors, encoded in $\phi \left(\omega \right)$, on the assimilation results can be analysed in more detail in the univariate case. This will allow us to gain an understanding of the relative order of magnitude of the different contributions. Let the error variances be *b* ^2^, *q* ^2^ and *r* ^2^ for background, model, and observational errors, respectively. We observe directly and the model is a constant *m* inside the assimilation window. A value of *m* = 0 maps any state variable to zero. Values in 0 < *m* < 1 constitute compressions of the state variable, the case *m* = 1 is the identity, and *m* > 1 is an expansion. Negative values have the same behaviour, but the difference is that they cause the state variable to alternate signs in consecutive time steps. Hence, we only consider *m* ≥ 0. We will analyse the results for the zero‐ and infinite‐memory cases, which apply for any $\phi \left(\omega \right)$. In the finite non‐zero *ω* cases, we have to specify the exact memory dependence. We restrict ourselves to exponential‐memory dependences here.

For the scalar case the model operators are just powers of *m*:

(41) $$M^{0:\tau }=\left[m^{\tau },m^{\tau -1},m^{\tau -2},\text{⋯}\;,m^{1},1\right].$$

The scalar versions of Equations 22, 28 and 30 are

(42) $$K_{x}^{0}=\frac{m^{\tau }b^{2}}{\gamma_{\tau }^{2}(m,\omega )},K_{v}^{j}=\frac{q^{2}}{\gamma_{\tau }^{2}(m,\omega )}\sum_{i=1}^{\tau }m^{\tau -i}\phi (|i-j|,\omega )$$

for *j* = {1,2,⋯,*τ*}, in which

(43) $$\gamma_{\tau }^{2}(m,\omega )=m^{2\tau }b^{2}+q^{2}\lambda_{\tau }^{2}(m,\omega )+r^{2},$$

and we used the notation:

(44) $$Q^{1:\tau }=q^{2}\Phi^{\tau },B^{\tau }=m^{2\tau }b^{2},\;\Lambda^{\tau }=q^{2}\lambda_{\tau }^{2}(m,\omega ).$$

The zero‐memory and infinite‐memory cases are given as

(45) $$\lim_{\omega \to 0}K_{v}^{j}\frac{q^{2}}{\gamma_{\tau }^{2}(m,0)}m^{\tau -j},\lim_{\omega \to \infty }K_{v}^{j}\frac{q^{2}}{\gamma_{\tau }^{2}(m,\infty )}\left(\frac{1-m^{\tau }}{1-m}\right).$$

Note that in the latter case the gain is exactly the same for all time steps.

The effect of the time‐correlation of model error resides in the factor

(46) $$\lambda_{\tau }^{2}(m,\omega )=\sum_{i=1}^{\tau }\sum_{j=1}^{\tau }m^{2\tau -(i+j)}\phi (|i-j|,\omega ).$$

Using $\sum_{j=1}^{J}ar^{j-1}=a(1-r^{J}){\left(1-r\right)}^{-1}$ (a property of geometric sums), the limits of zero‐memory and infinite‐memory of Equation 46 become

(47) $$\lim_{\omega \to 0}\lambda_{\tau }^{2}=\frac{1-m^{2\tau }}{1-m^{2}}\;\leq \;\lim_{\omega \to \infty }\lambda_{\tau }^{2}=\frac{{\left(1-m^{\tau }\right)}^{2}}{{\left(1-m\right)}^{2}},$$

where clearly the second expression is larger than the first, except for *τ* = 1 in which case $\lambda_{\tau }^{2}(m,\omega )=1$ in both limits. As function of *m* we have:

- When *m*→0, $\lambda_{\tau }^{2}(m,0)\to 1$ and $\lambda_{\tau }^{2}(m,\infty )\to 1$.
- When *m*→1, $\lambda_{\tau }^{2}(m,0)\to \tau$ and $\lambda_{\tau }^{2}(m,\infty )\to \tau^{2}$, i.e. the increase in memory leads to an increase of the compound model error variance.
- When *m*→*∞* (so *m* large), $\lambda_{\tau }^{2}(m,\omega )\to \infty$ and the leading‐order term is *m* ^2(*τ* − 1)^.

For an exponentially decaying memory, we use *Wolfram Mathematica* to get a closed‐form expression

(48) $$\begin{array}{ll} & \lambda_{\tau ,exp}^{2}(m,\omega ,\tau )\;\\ & \;=\frac{e^{1/\omega }\left(2m^{\tau }e^{\tau /\omega }-m^{2\tau }-1+2m\left(\frac{m^{2\tau }-1}{m^{2}-1}\right)\sinh(1/\omega )\right)}{\left(e^{1/\omega }-m)(me^{1/\omega }-1\right)}.\; \end{array}$$

**Figure 2:**
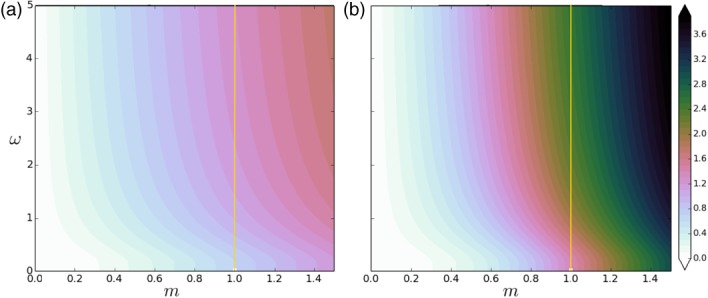
Effective contribution (as a multiple of q ^2^) of the model error to the total covariance at the time of the observation for an assimilation window of (a) τ = 2 and (b) τ = 4 time steps. This is shown for an univariate setting, and as function of the model m (horizontal axis) and the auto‐correlation memory ω of the model error (vertical axis), in the case an exponential memory. The value m = 1 is shown with a yellow line. Note that we are plotting the logarithm of λ ^2^(m,ω) [Colour figure can be viewed at wileyonlinelibrary.com]

The natural logarithm of Equation 48 is plotted in Figure 2 as a function of *m* (horizontal axis) and *ω* (vertical axis) for (a) *τ* = 2 and (b) *τ* = 4. The vertical yellow line indicates *m* = 1. It is clear that $\lambda_{\tau ,exp}^{2}$ grows as *τ* grows. For a fixed *ω*, $\lambda_{exp}^{2}$ grows exponentially as *m* grows. For a fixed *m*, $\lambda_{exp}^{2}$ grows as *ω* grows – as expected from Equation 47 – but the growth is slow. In fact, there is a sharp transition around *ω* = 1 for *m* ≤ 1. In contrast, for *m* > 1 the effect of *ω* on $\lambda_{exp}^{2}$ is smaller. Evaluating Equation 48 for *m* = 1 renders an undetermined form (0÷0) and one must take a limit. For the exponential‐memory case, we substitute $\lambda_{\tau ,exp}^{2}$ from Equation 48 into Equation 42 to get

(49) $$\begin{array}{ll} K_{v,exp}^{j}=\frac{q^{2}}{\gamma_{\tau ,exp}^{2}}\left(\frac{e^{(j-\tau )/\omega }}{1-e^{1/\omega }m}-\frac{m^{\tau }e^{(1-j)/\omega }}{e^{1/\omega }-m}\right. & \;\\ \left.-\frac{m^{\tau +1-j}(e^{2/\omega }-1)}{\left(e^{1/\omega }-m)(1-e^{1/\omega }m\right)}\right). & \; \end{array}$$

When the solution is expressed in terms of the state variables, we need the scalar version of Equation 37 – i.e. the gain acting on *x* ^*t*^– which now becomes

(50) $$K_{x}^{t}=\frac{m^{\tau +t}b^{2}}{\gamma_{\tau }^{2}(m,\omega )}+\frac{q^{2}}{\gamma_{\tau }^{2}(m,\omega )}\sum_{j=1}^{t}\sum_{i=1}^{\tau }m^{\tau +t-(i+j)}\phi (|i-j|,\omega ),$$

which has the following two limits:

(51) $$\begin{array}{ccc} \lim_{\omega \to 0}K_{x}^{t} & =\frac{m^{\tau +t}b^{2}}{\gamma_{\tau }^{2}(m,0)}+\frac{q^{2}}{\gamma_{\tau }^{2}(m,0)}\frac{m^{\tau -t}(1-m^{2t})}{1-m^{2}}, & \\ \lim_{\omega \to \infty }K_{x}^{t} & =\frac{m^{\tau +t}b^{2}}{\gamma_{\tau }^{2}(m,\infty )}+\frac{q^{2}}{\gamma_{\tau }^{2}(m,\infty )}\frac{\left(1-m^{t})(1-m^{\tau }\right)}{{\left(1-m\right)}^{2}}. \end{array}$$

and substituting again $\lambda_{\tau ,exp}^{2}$ from Equation 48 into Equation 37 we have explicitly

(52) $$\begin{array}{ccc} K_{x,exp}^{t}= & \frac{m^{\tau +t}b^{2}}{\gamma_{\tau ,exp}^{2}}+\frac{q^{2}e^{1/\omega }\left\{m^{t}\left(e^{-\tau /\omega }+e^{-t/\omega }\right)-e^{(t-\tau )/\omega }-m^{2t}\right\}}{\gamma_{\tau ,exp}^{2}(e^{1/\omega }-m)(e^{1/\omega }m-1)} & \\ & +\frac{q^{2}e^{1/\omega }\left\{m^{2t+1}\sinh(1/\omega )+m(1-e^{2/\omega })e^{-(2t+1)/\omega }\right\}}{\gamma_{\tau ,exp}^{2}(e^{1/\omega }-m)(e^{1/\omega }m-1)(m^{2}-1)}. \end{array}$$

In the univariate case, 0 ≤ *K* ≤ 1 (for all gains). As *K* grows, the influence of the observation on the analysis increases.

**Figure 3:**
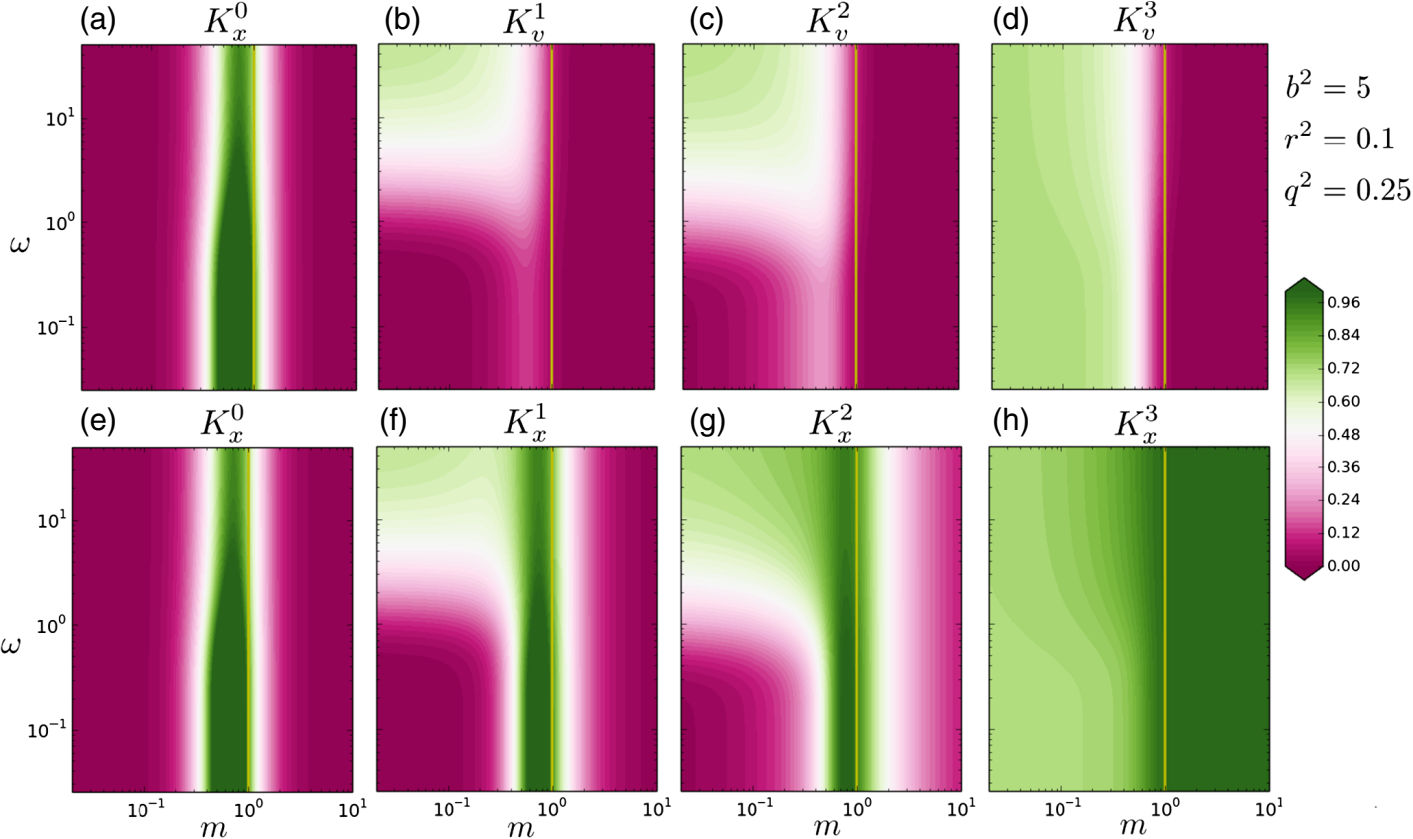
Impact on the model error of the assimilation of an observation at the end of an assimilation window of τ = 3 time steps for the univariate case, a model m (horizontal axes) and an auto‐correlation memory ω (vertical axes). These results correspond to the exponential memory case. (a) to (d) show the gains for the initial condition, and for the different model jumps. (e) to (h) show the gains for the initial conditions and the actual state variables at the different times. By construction, in this case the gains must be between 0 and 1. For gain values closer to 0 the analysis is closer to the background, while for gain values closer to 1 the analysis is closer to the observations [Colour figure can be viewed at wileyonlinelibrary.com]

For illustration we choose an assimilation window with *τ* = 3 time steps and variances *b* ^2^ = 5, *q* ^2^ = 0.25 and *r* ^2^ = 0.1. In Figure 3 we plot the gains (a–d) $K_{v,exp}^{j}$ and (e–h) $K_{x,exp}^{t}$. Each column shows a different time step from (a, e) *t* = 0 to (d, h) *t* = 3. In each panel we plot the gain for every combination of *m* (horizontal axis) and *ω* (vertical axis), with both axes plotted in logarithmic scale. In green areas the observation has more influence on the analysis than the background, and in pink areas the opposite happens.

Figure 3 reveals several properties:(a) The effect of observations is more efficiently communicated to past time‐steps when the memory *ω* is large. (b) The largest impact of the observation occurs at the observation time. (c) We see a different behaviour for compressive *m* < 1 and expansive *m* > 1 models, and for those close to persistence with *m* = 1.

**Figure 4:**
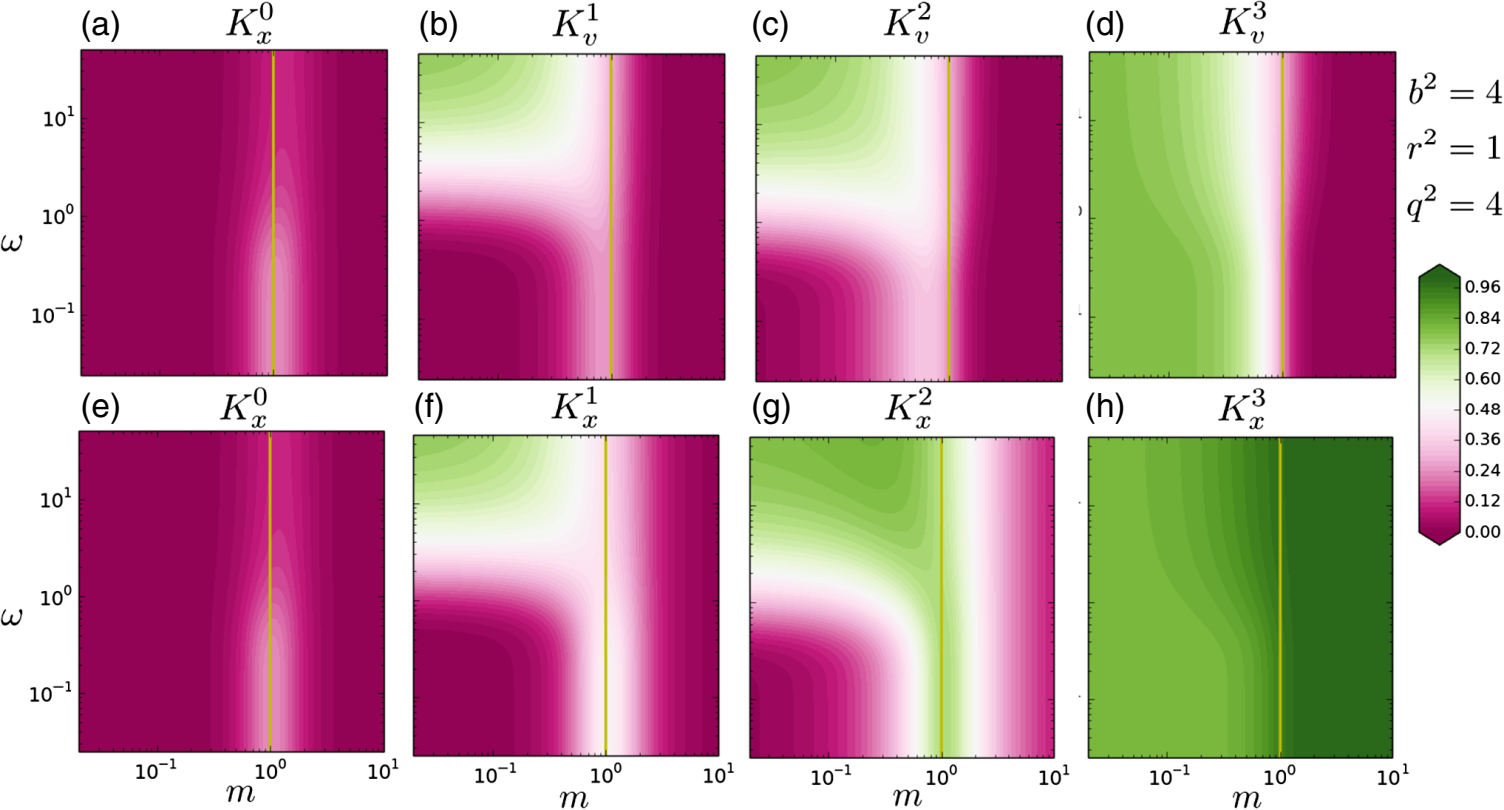
As Figure 3, but with different values for the background‐error, model‐error, and observational‐error covariances. Since r ^2^ increased, the observation has a lesser impact on the initial conditions and state variables at intermediate time steps [Colour figure can be viewed at wileyonlinelibrary.com]

We investigated different combinations of values for the variances, and the general behaviour was similar. For example, in Figure 4 we plot the case for *b* ^2^ = 4, *q* ^2^ = 4 and *r* ^2^ = 1. Since we increased the observational variance, the impact of the observation is smaller on all panels.

#### 2.4.1 Analysing the gains

**Figure 5:**
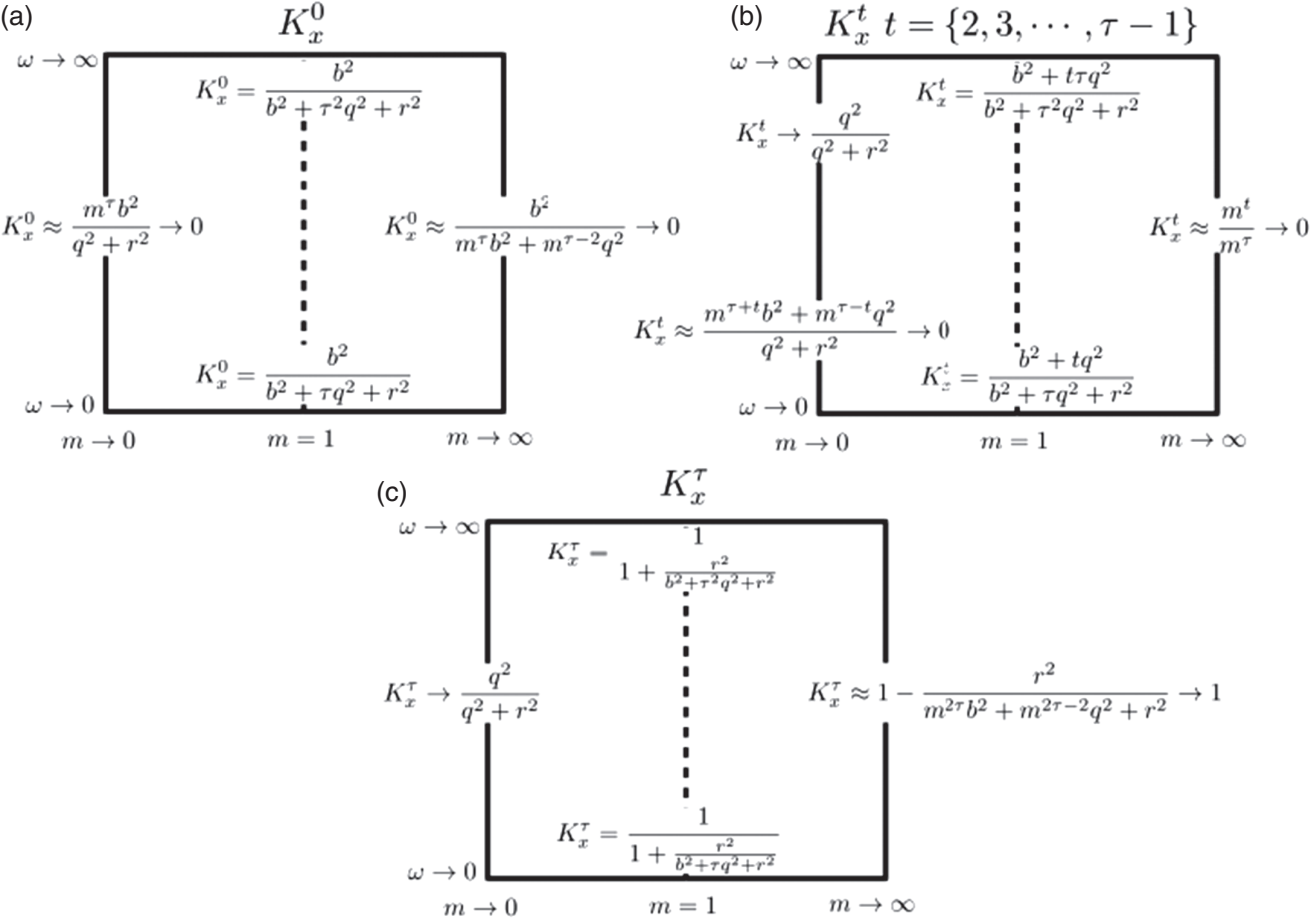
Schematic showing some limiting values of $K_{x}^{t}$, i.e. the impact of the observation in the model variables (a) at the initial time, (b) at some intermediate time in the assimilation window, and (c) at the end of the assimilation window, as a function of the model m (horizontal axes) and the autocorrelation memory of the model error ω (vertical axis). This figure explains qualitatively the behaviour of Figures 3 and 4, but the limiting values shown here are valid for a general temporal‐correlation function

In the final section on the univariate case, we shed some more light on the behaviour of the gains $K_{x}^{0}$, $K_{x}^{t}\;(0<t<\tau )$ and $K_{x}^{\tau }$. The summary of this analysis is given in Figure 5, which also includes the limiting expressions of these gains as *m*, *ω* and *τ* change.

These analytical results on the dependence on *m* can be understood as follows. Schematically, we can write

(53) $$K_{x}^{t}=\frac{Cov(t,\tau )}{Cov(\tau ,\tau )+R}.$$

In general, the numerator of $K_{x}^{t}$ arises from the covariance between the state at time *t* and the state at observation time *τ*. This covariance consists of two terms, the propagation of the background covariance from time 0, and the accumulated propagated model errors. The denominator consists of the total propagated state error at observation time, and the observation errors. The former consists again of a part related to the background covariance at time 0 propagated to time *τ*, and the accumulated propagated model errors.

For small *m*, most terms in the numerator will be small, since they are proportional to some positive power of *m*. There is one model error term that is not propagated, and that term is proportional to *ϕ*(|*τ* − *t*|,*ω*), which is small for *ω* < 1. The denominator is dominated by terms that do not contain propagation, specifically the observation error and the model error in the last model time step. Combining numerator and denominator, we see directly that the gain will be small. This means that observations at the end of a window have small influence on the state at other times when *m* is small. This is simply because the propagated state covariance will be very small at observation time, so the propagated state is much more accurate than the observation. An exception is when *ω* is large, in which case *ϕ*(|*τ* − *t*|,*ω*)→1, and the propagated error of the state is not small compared to the observation, so the observation can have an influence on the state, as shows in the figures.

For large *m*, the largest terms in the expression for $K_{x}^{t}$ are those that contain the propagation of the background covariance all the way to the observation time. The term in the numerator has *m* to the power *τ* + *t*, but the denominator has *m* to the power 2*τ*, and hence, again,the gain will be small. This means that observations at the end of a window have small influence on the state at other times when *m* is large. Although the observation is much more accurate than the propagated state, the propagation of the innovation backwards in time, i.e. in the direction where the model contracts, leads to a small influence.

## 3 INEXACT TIME CORRELATION AND FINITE ENSEMBLE SIZE

We have discussed the effect of temporally correlated model error has on a time window of the Kalman smoother. In this section we consider the effect of two sources of imperfection: an incorrectly prescribed memory time‐scale for the model error, and covariance information coming from samples with a finite‐ensemble size.

### 3.1 Errors from inexact time autocorrelation

Assume that we know **Q** exactly, but we do not know the real memory time‐scale *ω*. Hence, the DA forecast model uses a *guessω*_*g*_, and the model error used in the DA is the result of this mis‐specification. While **Φ** ^1:*τ*^ has functions *ϕ*(|*i* − *j*|,*ω*), the guess $\Phi_{g}^{1:\tau }$ has functions *ϕ*(|*i* − *j*|,*ω*_*g*_). Their difference $\epsilon_{\Phi }^{1:\tau }\in R^{\tau \times \tau }$ is

(54) $$\epsilon_{\Phi }^{1:\tau }=\Phi_{g}^{1:\tau }-\Phi^{1:\tau }$$

and

(55) $$Q_{g}^{1:\tau }-Q^{1:\tau }=\epsilon_{\Phi }^{1:\tau }⊗Q.$$

**Figure 6:**
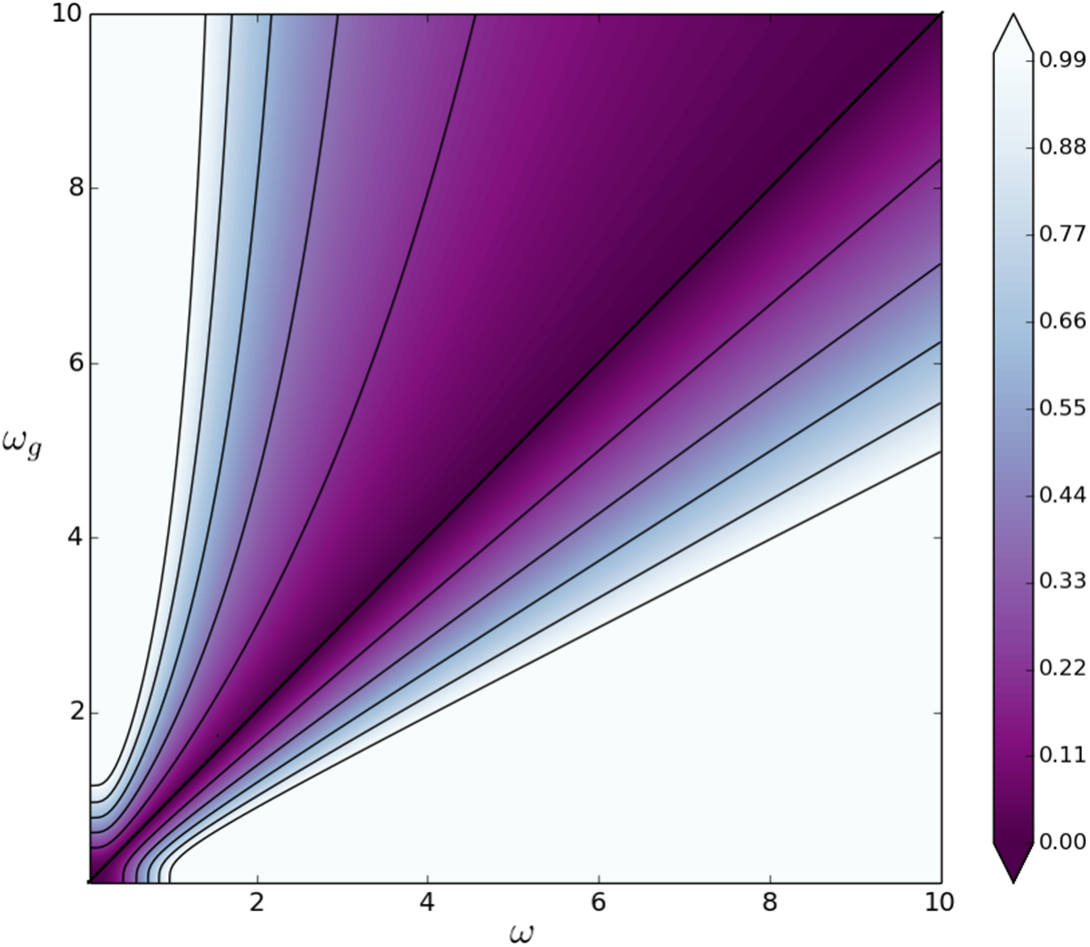
Relative magnitude of the difference between a guess model‐error temporal‐correlation matrix $\Phi_{\omega }^{1:\tau }$, and the real model‐error temporal‐correlation matrix **Φ** ^1:τ^ in the exponential memory case for an assimilation window of size τ = 10. The largest eigenvalue is shown as a function of the real memory ω (horizontal axis) and the guess memory ω_g_ (vertical axis). The solid lines are the contours: {0,0.2,0.4,0.6,0.8,0.1}. Note that the values do not only depend on the difference ω_g_ − ω [Colour figure can be viewed at wileyonlinelibrary.com]

We need a measure of the “magnitude” of the difference $\epsilon_{\Phi }^{1:\tau }$ relative to the “magnitude” of **Φ** ^1:*τ*^. We use the metric

(56) $$\sigma_{\epsilon_{\phi }}^{2}=\|\epsilon_{\Phi }^{1:\tau }{\left(\Phi^{1:\tau }\right)}^{-1}\|,$$

where the norm ‖.‖ is the maximum eigenvalue. Finding the analytical dependence of Equation 56 on *ω* and *ω*_*g*_ is not simple. Hence, we have resorted to a numerical experiment to illustrate the behaviour of $\sigma_{\epsilon_{\phi }}^{2}\left(\omega ,\omega_{g},\tau \right)$ as function of the mis‐specification of the memory term. Results are shown in Figure 6 for an assimilation window of size *τ* = 10, using an exponential‐memory function. This figure is generated in the following manner:

- We choose an *ω* to produce a matrix **Φ** ^1:*τ*^.
- We choose an *ω*_*g*_ to produce a *guess* matrix $\Phi_{g}^{1:\tau }$.
- We evaluate Equations 54 and 56. The resulting value is saved.
- We repeat these steps for every pair $\left(\omega ,\omega_{g}\right)$, and we populate a matrix.

The resulting matrix is plotted for different values of *ω* (horizontal axis) and of *ω*_*g*_ (vertical axis). The colour bar spans the interval 0 (dark purple) to 1 (light blue), and everything above 1 is plotted in white. Black lines show the contours corresponding to $\sigma_{\epsilon_{\phi }}^{2}=\{0,0.2,0.4,0.6,0.8,1.0\}$. This figure shows that having *ω*_*g*_ < *ω* results in larger $\sigma_{\epsilon_{\phi }}^{2}$ than the contrary situation. Hence underestimating the temporal correlation time‐scale leads to larger errors then overestimating it. In general, there is a large region in which the magnitude is smaller than unity. The value *does not simply* depend on the difference |*ω* − *ω*_*g*_| or the ratio *ω*/*ω*_*g*_, but their individual values as well. We performed experiments with different values of *τ* and they yielded similar results, so they are not included.

The corresponding analysis mean and covariance react to the mis‐specification of the memory scale as follows. Interestingly, both **z** ^0:*τ*,*b*^ and $z_{g}^{0:\tau ,b}$ share the same expectation:

(57) $$E\left[z^{0:\tau ,b}\right]=E\left[z_{g}^{0:\tau ,b}\right]=\mu_{z}^{0:\tau ,b}$$

and the only difference is in the covariances:

(58) $$D_{g}^{0:\tau }=Cov\left[z_{g}^{0:\tau ,b}\right]=\left[\begin{array}{cc} B & 0\\ 0 & Q_{g}^{1:\tau } \end{array}\right]$$

and the rest of the problem is the same. This means that we have the posterior pdf:

(59) $$z_{g}^{0:\tau ,a}|y∼N\left(\mu_{z,g}^{0:\tau ,a},A_{z,g}^{0:\tau }\right)$$

with $\mu_{z,g}^{0:\tau ,a}\in R^{(1+\tau )N_{x}}$ and $A_{z,g}^{0:\tau }\in R^{(1+\tau )N_{x}\times (1+\tau )N_{x}}$ defined as:

(60) $$\begin{array}{ccc} \mu_{z,g}^{0:\tau ,a} & =\left(I-K_{z,g}^{0:\tau }HM^{0:\tau }\right)\mu_{z}^{0:\tau ,b}+K_{z,g}^{0:\tau }y, & \\ A_{z,g}^{0:\tau } & =\left(I-K_{z,g}^{0:\tau }HM^{0:\tau }\right)D_{g}^{0:\tau }. \end{array}$$

with

(61) $$\begin{array}{ccc} \Gamma_{g}^{\tau } & =HM^{0:\tau }D_{g}^{0:\tau }{\left(M^{0:\tau }\right)}^{T}H^{T}+R, & \\ K_{z,g}^{0:\tau } & =D_{g}^{0:\tau }{\left(M^{0:\tau }\right)}^{T}H^{T}{\left(\Gamma_{g}^{\tau }\right)}^{-1}. \end{array}$$

Therefore, substituting **Φ** ^1:*τ*^ with $\Phi_{g}^{1:\tau }$ renders a different $D_{g}^{0:\tau }$, $\Gamma_{g}^{\tau }$ and $K_{z,g}^{0:\tau }$, and these are combined in a nonlinear fashion to produce guess analysis solutions. The differences with the exact solutions in Equation 12 are not simple to analyse. Before doing so let us first consider the noise introduced by finite‐size ensembles.

### 3.2 Direct and indirect errors coming from ensemble statistics

Consider an ensemble of *N*_*e*_ elements sampled from the background pdf $N\left(\mu_{z}^{0:\tau ,b},D^{0:\tau }\right)$. The *n*_*e*_th member is

(62) $$z_{n_{e}}^{0:\tau ,b}=\left[\begin{array}{c} x_{n_{e}}^{0,b}\\ v_{n_{e}}^{1:\tau ,b} \end{array}\right]$$

One can generate a sample $\eta_{n_{e}}$ from the observational error pdf $N\left(0,R\right)$. Following Burgers *et al.* (1999), but perturbing the observational departure from the background instead of the observations for statistical consistency, we find

(63) $$d_{n_{e}}=y-(Hx_{n_{e}}^{\tau }+\eta_{n_{e}})$$

where we have again, but now for each ensemble member $x_{n_{e}}^{\tau }=M^{0:\tau }z_{n_{e}}^{0:\tau ,b}$. One can then apply Equation 19 using the sample expressions (62) and (63) to get

(64) $$z_{n_{e}}^{0:\tau ,a}=z_{n_{e}}^{0:\tau ,b}+K_{z}^{0:\tau }d_{n_{e}}.$$

Note that Equation 64 uses the exact gain $K_{z}^{0:\tau }$. The associated *n*_*e*_th cost function uses the true covariance *D* ^0:*τ*^:

(65) $$\begin{array}{ll} & J_{n_{e}}(z^{0:\tau })\;\\ & =\frac{1}{2}{\left(z^{0:\tau }-z_{n_{e}}^{0:\tau ,b}\right)}^{T}{\left(D^{0:\tau }\right)}^{-1}\left(z^{0:\tau }-z_{n_{e}}^{0:\tau ,b}\right)\;\\ & +\frac{1}{2}{\left\{y-\left(HM^{0:\tau }z^{0:\tau }+\eta_{n_{e}}\right)\right\}}^{T}R^{-1}\left\{y-\left(HM^{0:\tau }z^{0:\tau }+\eta_{n_{e}}\right)\right\}.\; \end{array}$$

A collection of *N*_*e*_ values constructed using Equation 64 constitute a sample from the posterior pdf $N\left(\mu_{z}^{0:\tau ,a},A_{z}^{0:\tau }\right)$, with the moments defined as in Equations 19 and 20. Clearly, the estimators coming from any finite‐size ensemble constructed in this way will have direct, i.e. Monte‐Carlo, sampling errors.

Now we discuss the more subtle indirect sampling errors. These come from using the sample estimator **B**_*e*_ instead of **B** in the computation of the analysis values. This is a natural step of the Kalman filter/smoother and it helps in incorporating flow‐dependent information since this covariance matrix usually evolves in time and it is often impossible to compute it exactly. In this case, the associated cost function is identical to Equation 65 but with **D** ^0:*τ*^ replaced by $D_{e}^{0:\tau }$. This block‐matrix is

(66) $$D_{e}^{0:\tau }=\left[\begin{array}{cc} B_{e} & 0\\ 0 & Q^{1:\tau } \end{array}\right]$$

This leads to an ensemble‐based total covariance

(67) $$\Gamma_{e}^{\tau }=HM^{0:\tau }D_{e}^{0:\tau }{\left(M^{0:\tau }\right)}^{T}H^{T}+R$$

and an ensemble‐based gain

(68) $$K_{z,e}^{0:\tau }=D_{e}^{0:\tau }{\left(M^{0:\tau }\right)}^{T}H^{T}{\left(\Gamma_{e}^{\tau }\right)}^{-1}.$$

Then one can construct each analysis member as

(69) $$z_{n_{e}}^{0:\tau ,a}=z_{n_{e}}^{0:\tau ,b}+K_{z,e}^{0:\tau }d_{n_{e}},$$

where we emphasize that the empirical gain $K_{z,e}^{0:\tau }$ has been used. An ensemble constructed in this manner will have both direct and indirect sampling errors. This indirect sampling error is taken into consideration, for instance, in the creation of the EnKF‐N of Bocquet *et al.* (2014). From now on, we will use $z_{n_{e}}^{0:\tau ,a}$ as defined in Equation 69 and not in Equation 64.

### 3.3 Effects of the two sources of imperfection

We now combine the two sources of imperfection and perform a more detailed examination if their consequences. From the last two subsections recall that the subindex g indicates variables related to the guess model error $Q_{g}^{1:\tau }$, whereas the subindex e indicates variables related to the sample covariance **B**_*e*_. It is clear that some elements will have two subindices, for example $K_{ge}^{0:\tau }$.

For each ensemble member we decompose $z_{g,n_{e}}^{0:\tau ,b}$ and $d_{g,n_{e}}$ as the sum of this expectation and a perturbation:

(70) $$\begin{array}{ccc} z_{g,n_{e}}^{0:\tau ,b} & =\mu_{z}^{0:\tau ,b}+\zeta_{g,n_{e}}^{0:\tau ,b} & \\ d_{g,n_{e}} & =d+\delta_{g,n_{e}} \end{array}$$

where $\zeta_{g,n_{e}}^{0:\tau ,b}$ is a sample from $N\left(0,D^{0:\tau }\right)$, **d** defined as in Equation 32 and the perturbation $\delta_{g,n_{e}}$ is

(71) $$\delta_{g,n_{e}}=-\left(HM^{0:\tau }\zeta_{g,n_{e}}^{0:\tau ,b}+\eta_{n_{e}}\right)$$

Hence, we can write the (imperfect) analysis value for each ensemble member $z_{g,n_{e}}^{0:\tau ,a}$ as:

(72) $$\underline{z_{g,n_{e}}^{0:\tau ,a}=\left(\mu_{z}^{0:\tau ,b}+K_{z,ge}^{0:\tau }d\right)+\left(\zeta_{g,n_{e}}^{0:\tau ,b}+K_{z,ge}^{0:\tau }\delta_{g,n_{e}}\right)}.$$

The first parenthesis is the update for the means, while the second parenthesis is the update for the perturbations. The *empirical* gain $K_{z,ge}^{0:\tau }$ appears in both. The structure of Equation 72 makes it difficult to disentangle the contributions from the two sources of error. To proceed we follow what van Leeuwen (1999) and Sacher and Bartello (2005) did for the EnKF. We express **D**_*ge*_ as a departure from the exact **D** ^0:*τ*^:

(73) $$D_{ge}^{0:\tau }=D^{0:\tau }+\epsilon_{D}^{0:\tau }=\left(I+\epsilon_{D}^{0:\tau }{\left(D^{0:\tau }\right)}^{-1}\right)D^{0:\tau }$$

Explicitly $\epsilon_{D}^{0:\tau }\in R^{(1+\tau )N_{x}\times (1+\tau )N_{x}}$ is the block‐matrix:

(74) $$\epsilon_{D}^{0:\tau }=\left[\begin{array}{cc} B_{e}-B & 0\\ 0 & Q_{g}^{1:\tau }-Q^{1:\tau } \end{array}\right]=\left[\begin{array}{cc} \epsilon_{B} & 0\\ 0 & \epsilon_{\Phi }^{1:\tau }⊗Q \end{array}\right]$$

The sample covariance **B**_*e*_ is a random matrix. Since **x** ^0,*b*^ has a MVG pdf, **B**_*e*_ follows a Wishart distribution with *N*_*x*_ − 1 degrees of freedom. $\epsilon_{B}\in R^{N_{x}\times N_{x}}$ is also a random matrix, but it can have both positive and negative values in its entries, and its distribution is not simple. $\epsilon_{\Phi }^{1:\tau }$ is not random since it comes from a wrong prescribed time‐scale *ω*_*g*_.

We can write the ratio $\epsilon_{D}^{0:\tau }{\left(D^{0:\tau }\right)}^{-1}$ as a block‐matrix :

(75) $$\epsilon_{D}^{0:\tau }{\left(D^{0:\tau }\right)}^{-1}=\left[\begin{array}{cc} \epsilon_{B}B^{-1} & 0\\ 0 & \left(\epsilon_{\Phi }^{1:\tau }{\left(\Phi^{1:\tau }\right)}^{-1}\right)⊗I_{N_{x}} \end{array}\right],$$

where we have used the mixed‐product property of the Kronecker product to get the bottom‐right element. This property states that if **A**, **B**, **C** and **D** are matrices of such size that one can form the matrix products **AC** and **BD**, then $\left(A⊗B\right)\left(C⊗D\right)=\left(AC\right)⊗\left(BD\right)$ (e.g. Golub and Loan, 1983; Horn and Johnson, 1991).

The sample‐based total covariance at the end of the assimilation window $\Gamma_{ge}^{\tau }$ is

(76) $$\Gamma_{ge}^{\tau }=\Gamma^{\tau }+\Gamma_{g\epsilon }^{\tau }=\left(I+\Gamma_{g\epsilon }^{\tau }{\left(\Gamma^{\tau }\right)}^{-1}\right)\Gamma^{\tau },$$

where $\Gamma_{g\epsilon }^{\tau }=HM^{0:\tau }\epsilon_{D}^{0:\tau }{\left(M^{0:\tau }\right)}^{T}H^{T}$ is the contribution from the sampling error. Finally the *ensemble‐based* gain $K_{z,e}^{0:\tau }$ can be written as

(77) $$\underline{K_{z,ge}^{0:\tau }=\left(I+\epsilon_{D}^{0:\tau }{\left(D^{0:\tau }\right)}^{-1}\right)K_{z}^{0:\tau }{\left(I+\Gamma_{g\epsilon }^{\tau }{\left(\Gamma^{\tau }\right)}^{-1}\right)}^{-1}}$$

Therefore the *ensemble gain* is a the *real gain* multiplied by two factors. The left factor contains the error in the construction of the covariance matrix **D**_*e*_, and the right factor contains the respective error in the total covariance $\Gamma_{ge}^{\tau }$ at the time of the observation.

### 3.4 Small error approximations

Every expression has been *exact* up to this point. To continue we require an approximation: we consider that the **B**_*e*_ is “not too far” from **B**, and that *ω*_*g*_ is “not too far” from *ω*. To be more precise:

(78) $$\|\epsilon_{D}^{0:\tau }{\left(D^{0:\tau }\right)}^{-1}\|\ll 1,\|\Gamma_{g\epsilon }^{\tau }{\left(\Gamma^{\tau }\right)}^{-1}\|\ll 1.$$

We perform a Taylor expansion for the right factor (inverse) in Equation 77, and use Equation 78 to neglect all terms after the linear:

(79) $${\left(I+\Gamma_{g\epsilon }^{\tau }{\left(\Gamma^{\tau }\right)}^{-1}\right)}^{-1}=I-\Gamma_{g\epsilon }^{\tau }{\left(\Gamma^{\tau }\right)}^{-1}+O\left(\|\Gamma_{g\epsilon }^{\tau }{\left(\Gamma^{\tau }\right)}^{-1}{\|}^{2}\right).$$

Substituting this into Equation 77 we have

(80) $$K_{z,rmge}^{0:\tau }\approx \left(I+\epsilon_{D}^{0:\tau }{\left(D^{0:\tau }\right)}^{-1}\right)K_{z}^{0:\tau }\left(I-\Gamma_{g\epsilon }^{\tau }{\left(\Gamma^{\tau }\right)}^{-1}.\right)$$

After performing the products and ignoring the term proportional to $\epsilon_{D}^{0:\tau }{\left(D^{0:\tau }\right)}^{-1}\Gamma_{g\epsilon }^{\tau }{\left(\Gamma^{\tau }\right)}^{-1}$, the empirical gain $K_{z,ge}^{0:\tau }$ can be approximately decomposed into the *sum* of two components:

(81) $$\underline{K_{z,ge}^{0:\tau }\approx K_{z}^{0:\tau }+K_{z,g\epsilon }^{0:\tau }}.$$

$K_{z}^{0:\tau }$ is the exact gain defined in Equation 21 and $K_{z,g\epsilon }^{0:\tau }$ is the gain arising from the errors (both from sampling and incorrect memory). Explicitly this is

(82) $$\underline{K_{z,g\epsilon }^{0:\tau }=\epsilon_{D}^{0:\tau }{\left(D^{0:\tau }\right)}^{-1}K_{z}^{0:\tau }-K_{z}^{0:\tau }\Gamma_{g\epsilon }^{\tau }{\left(\Gamma^{\tau }\right)}^{-1}}.$$

$K_{z,g\epsilon }^{0:\tau }$ has two terms; both are contractions of the real gain $K_{z}^{0:\tau }$ by factors $\epsilon_{D}^{0:\tau }{\left(D^{0:\tau }\right)}^{-1}$ and $\Gamma_{g\epsilon }^{\tau }{\left(\Gamma^{\tau }\right)}^{-1}$ respectively. For the latter ratio we have:

(83) $$\begin{array}{ll} & \Gamma_{g\epsilon }^{\tau }{\left(\Gamma^{\tau }\right)}^{-1}\;\\ & =\left(HM^{0:\tau }\epsilon_{D}^{0:\tau }{\left(M^{0:\tau }\right)}^{T}H^{T}\right){\left(HM^{0:\tau }D^{0:\tau }{\left(M^{0:\tau }\right)}^{T}H^{T}+R\right)}^{-1}, \end{array}$$

so for general **H**, it is not trivial to determine which of the two terms in Equation 82 is larger.

Substituting Equation 81 into Equation 72 yields an approximate expression for the analysis value of each ensemble member. It it formed of three terms:

(84) $$\underline{z_{g,n_{e}}^{0:\tau ,a}\approx \mu_{z}^{0:\tau ,a}+\zeta_{g,n_{e}}^{0:\tau ,a}+K_{z,g\epsilon }^{0:\tau }\left(d+\delta_{g,n_{e}}\right)}.$$

The first term in Equation 84 is the exact analysis mean:

(85) $$\mu_{z}^{0:\tau ,a}=\mu_{z}^{0:\tau ,b}+K_{z}^{0:\tau }d,$$

which is equivalent to Equation 19. The second term is the *direct* sampling error:

(86) $$\zeta_{g,n_{e}}^{0:\tau ,a}=\zeta_{g,n_{e}}^{0:\tau ,b}+K_{z}^{0:\tau }\delta_{g,n_{e}},$$

which is necessary to have correct probabilistic characteristics for the ensemble. $\zeta_{g,n_{e}}^{0:\tau ,a}$ is a realization of a random variable with pdf $N\left(0,A_{z,g}^{0:\tau }\right)$. The *guess* analysis covariance is

(87) $$A_{z,g}^{0:\tau }=\left(I-K_{z}^{0:\tau }HM^{0:\tau }\right)D_{g}^{0:\tau }.$$

$A_{z,g}^{0:\tau }$ contains the exact gain, which means that the reduction in uncertainty due to the DA step is correct. The only source of error is the incorrect time‐scale *ω*_*g*_ in $D_{g}^{0:\tau }$. Finally we have the *indirect* sampling errors

(88) $$K_{z,g\epsilon }^{0:\tau }\left(d+\delta_{g,n_{e}}\right),$$

which are formed of two terms: one linear and one a nonlinear product.

These expressions allow us to calculate the imperfections in the analysis mean and covariance. Since both $K_{z,ge}^{0:\tau }$ and $K_{z,g\epsilon }^{0:\tau }$ are constant for all members of a given ensemble, the sample mean is given by

(89) $$\underline{{\bar{z}}^{0:\tau ,a}=\mu_{z}^{0:\tau ,a}+{\bar{\zeta }}_{g}^{0:\tau ,a}+K_{z,g\epsilon }^{0:\tau }\left(d+{\bar{\delta }}_{g}\right)},$$

where the overbar denotes the arithmetic average. The sample covariance $A_{z,ge}^{0:\tau }=Cov_{N_{e}}\left(z_{g,1:N_{e}}^{0:\tau ,a}\right)$ is more complicated to compute. In a finite‐size sample the observational‐error covariance **R**_*e*_ = **R**+***ρ*** is not exact – this comes from perturbing **Hx** ^*τ*^ in Equation 63 – and spurious correlations between state variables and observations $\Sigma_{ge}={Cov}_{N_{e}}\left(z_{g,1:N_{e}}^{0:\tau ,b},y_{1:N_{e}}\right)$ can arise. The full expression for $A_{z,ge}^{0:\tau }$ is:

(90) $$\begin{array}{ll} A_{z,ge}^{0:\tau }= & \left(I-K_{z,ge}^{0:\tau }HM^{0:\tau }\right)(D^{0:\tau }+\epsilon_{D}^{0:\tau })\left(I-K_{z,ge}^{0:\tau }HM^{0:\tau }\right)\;\\ & +K_{z,ge}^{0:\tau }(R+\rho ){\left(K_{z,ge}^{0:\tau }\right)}^{T}+\left(I-K_{z,ge}^{0:\tau }HM^{0:\tau }\right)\Sigma_{ge}{\left(K_{z,ge}^{0:\tau }\right)}^{T}\;\\ & +K_{z,ge}^{0:\tau }\Sigma_{ge}{\left(I-K_{z,ge}^{0:\tau }HM^{0:\tau }\right)}^{T}.\; \end{array}$$

The inexact **R**_*e*_ *does not* participate in the gain; it only appears in the direct sampling effect. We consider that we have either a second‐order exact sampling scheme (Pham, 2002) or a large‐enough sample such that both ***ρ***→**0** and **Σ**_*ge*_→**0**. With these assumptions, several terms in Equation 90 are null (or at least negligible). If we take the remaining terms, substitute $K_{z,ge}^{0:\tau }$ from Equations 81 and 82, and keep only leading‐order terms, we can express $A_{z,ge}^{0:\tau }$ approximately as a departure from the true **A** ^0:*τ*^:

(91) $$\underline{A_{z,ge}^{0:\tau }\approx A_{z}^{0:\tau }+A_{z,g\epsilon }^{0:\tau }},$$

with the exact part found in Equation 20, and the error part is

(92) $$\begin{array}{ll} & \underline{A_{z,g\epsilon }^{0:\tau }=\left(I-K_{z}^{0:\tau }HM^{0:\tau }\right)\epsilon_{D}^{0:\tau }-K_{z,g\epsilon }^{0:\tau }HM^{0:\tau }D^{0:\tau }}\;\\ & \underline{-\epsilon_{D}^{0:\tau }{\left(M^{0:\tau }\right)}^{T}H^{T}{\left(\Gamma^{\tau }\right)}^{-1}HM^{0:\tau }\epsilon_{D}^{0:\tau }}.\; \end{array}$$

The first term in Equation 92 is direct sampling error. It corresponds to the reduction of $\epsilon_{D}^{0:\tau }$ due to the action of the exact gain. The second and third terms are indirect sampling noise. The second term is the a reduction of **D** ^0:*τ*^ due to the use of the inexact part of the gain, and it is linear in $\epsilon_{D}^{0:\tau }$. The last term is quadratic in $\epsilon_{D}^{0:\tau }$ and is called in‐breeding, to be discussed later.

### 3.5 Behaviour of the sample estimators

Table 1 summarizes the additive elements of the approximate ensemble analysis mean and covariance in the small error approximation. We separate the exact part and the direct and indirect sampling errors.

**Table 1:** Additive elements of the approximate ensemble analysis mean and covariance in the small error approximation

	Exact	Direct sampling	Indirect sampling	Indirect sampling
	part	error	error (linear)	error (nonlinear)
${\bar{z}}_{ge}^{0:\tau ,a}$	$\mu_{z}^{0:\tau }$	$+{\bar{\zeta }}_{g}^{0:\tau ,a}$	$+K_{z,g\epsilon }^{0:\tau }d$	$+K_{z,g\epsilon }^{0:\tau }{\bar{\delta }}_{g}$
$A_{z,ge}^{0:\tau }$	$A_{z}^{0:\tau }$	$+\left(I-K_{z}^{0:\tau }HM^{0:\tau }\right)\epsilon_{D}^{0:\tau }$	$-K_{z,g\epsilon }^{0:\tau }HM^{0:\tau }D^{0:\tau }$	$-{\left(HM^{0:\tau }\epsilon_{D}^{0:\tau }\right)}^{T}{\left(\Gamma^{\tau }\right)}^{-1}HM^{0:\tau }\epsilon_{D}^{0:\tau }$

Both estimators have an exact part and errors coming from both sampling (direct and indirect) and the mis‐specification of the memory. The errors arising from $\epsilon_{\Phi }^{0:\tau }$ are not random, and hence do not depend on sample size. The random variables ${\bar{\zeta }}_{g}^{0:\tau ,a}$ and ${\bar{\delta }}_{g}$ have zero expected value but incorrect covariances $A_{z,g}^{0:\tau }/N_{e}$ and $\Gamma_{g}^{\tau }/N_{e}$ respectively due to the guess for the temporal correlations denoted by g. The presence of $K_{z,g\epsilon }^{0:\tau }d$ causes a bias because of the dependence on $\epsilon_{\Phi }^{0:\tau }$. If ***ϵ***_*B*_ were not random, but instead were fixed as a result of an incorrect static estimator of **B**, it would have similar consequences as $\epsilon_{\Phi }^{0:\tau }$.

In the last part of this section we consider $\epsilon_{\Phi }^{0:\tau }=0$ (correct memory), and focus only on the behaviour of *random* errors coming from ***ϵ***_*B*_ as *N*_*e*_ grows. We take the expected value

(93) $$E\left[{\bar{z}}^{0:\tau ,a}{|}_{\omega_{g}=\omega }\right]=\mu_{z}^{0:\tau ,a}+E\left[{\bar{\zeta }}^{0:\tau ,a}\right]+E\left[K_{z,\epsilon }^{0:\tau }\right]d+E\left[K_{z,\epsilon }^{0:\tau }\bar{\delta }\right].$$

The first term in Equation 93 is the exact value, and the second has zero expected value $E\left[{\bar{\zeta }}^{0:\tau ,a}\right]=0\in R^{(\tau +1)N_{x}}$. The third term also has zero expected value. This can be seen by writing explicitly:

(94) $$E\left[K_{z,\epsilon }^{0:\tau }\right]=E\left[\epsilon_{D}^{0:\tau }\right]{\left(D^{0:\tau }\right)}^{-1}K_{z}^{0:\tau }-K_{z}^{0:\tau }E\left[\Gamma_{\epsilon }^{\tau }\right]{\left(\Gamma^{\tau }\right)}^{-1},$$

where both $E\left[\epsilon_{D}^{0:\tau }\right]=0\in R^{(1+\tau )N_{x}\times (1+\tau )N_{x}}$ and $E\left[\Gamma_{\epsilon }^{\tau }\right]=0\in R^{N_{y}\times N_{y}}$. For the latter we can see this explicitly via:

(95) $$E\left[\Gamma_{\epsilon }^{\tau }\right]=HM^{0:\tau }\left[\begin{array}{cc} E\left[\epsilon_{B}\right] & 0\\ 0 & 0 \end{array}\right]{\left(M^{0:\tau }\right)}^{T}H^{T}=0\in R^{N_{y}\times (1+\tau )N_{x}}.$$

Finally, we are left with the expected value of the nonlinear product $E\left[K_{z,\epsilon }^{0:\tau }\bar{\delta }\right]$:

(96) $$E\left[K_{z,\epsilon }^{0:\tau }\bar{\delta }\right]=-E\left[K_{z,\epsilon }^{0:\tau }HM^{0:\tau }{\bar{\zeta }}^{0:\tau ,b}\right]-E\left[K_{z,\epsilon }^{0:\tau }\bar{\eta }\right].$$

The second term is zero since $K_{z,\epsilon }^{0:\tau }$ and $\bar{\eta }$ are statistically independent. Hence

(97) $$E\left[K_{z,\epsilon }^{0:\tau }\bar{\eta }\right]=E\left[K_{z,\epsilon }^{0:\tau }\right]E\left[\bar{\eta }\right]=0.$$

We can write the first term of (96) as:

(98) $$\begin{array}{ll} & E\left[K_{z,\epsilon }^{0:\tau }HM^{0:\tau }{\bar{\zeta }}^{0:\tau ,b}\right]\;\\ & \;=E\left[\left(\epsilon_{D}^{0:\tau }{\left(D^{0:\tau }\right)}^{-1}K_{z}^{0:\tau }-K_{z}^{0:\tau }\Gamma_{\epsilon }^{\tau }{\left(\Gamma^{\tau }\right)}^{-1}\right)HM^{0:\tau }{\bar{\zeta }}^{0:\tau ,b}\right], \end{array}$$

which is not an easy expression. An application of Basu's theorem (Basu, 19551955) states that if $\bar{z}$ and **Σ**_*z*_ are the sample mean and sample covariance coming from a MGD, they are statistically independent (e.g. Ghosh, 2002). Hence the expected value of their product is the product of their expected values. However, in Equation 98 we have the expected value of products involving transformations of both ${\bar{\zeta }}^{0:\tau ,b}$ and **B**_*e*_, so the validity of independence may depend on the particular structure of the matrices involved.

After the previous examination, we can finally state that

(99) $$\underline{E\left[{\bar{z}}^{0:\tau ,a}{|}_{\omega_{g}=\omega }\right]=\mu_{z}^{0:\tau }-E\left[K_{z,\epsilon }^{0:\tau }HM^{0:\tau }{\bar{\zeta }}^{0:\tau ,b}\right]},$$

which implies that small sampling errors can only produce bias through the nonlinear product $K_{z,\epsilon }^{0:\tau }HM^{0:\tau }{\bar{\zeta }}^{0:\tau ,b}$. According to the knowledge of the authors, this has not been noticed in the literature before, and is also true for ensemble Kalman filters when the time dimension is disregarded. In the experiments discussed in the next section we show that this effect can be substantial, and much larger than the estimated error covariances.

The expected value of the sample analysis covariance is:

(100) $$\begin{array}{ll} E\left[A_{e}^{z}{|}_{\omega_{g}=\omega }\right]= & A_{z}^{0:\tau }+\left(I-K_{z}^{0:\tau }HM^{0:\tau }\right)E\left[\epsilon_{D}^{0:\tau }\right]\;\\ & -E\left[K_{z,\epsilon }^{0:\tau }\right]HM^{0:\tau }D^{0:\tau }\;\\ & -E\left[\epsilon_{D}^{0:\tau }{\left(M^{0:\tau }\right)}^{T}H^{T}{\left(\Gamma^{\tau }\right)}^{-1}HM^{0:\tau }\epsilon_{D}^{0:\tau }\right].\; \end{array}$$

We know that both $E\left[\epsilon_{D}^{0:\tau }\right]=0$ and $E\left[K_{z,\epsilon }^{0:\tau }\right]=0$. However, the last term is quadratic in $\epsilon_{D}^{0:\tau }$ so its expected value *is not* zero. Therefore

(101) $$\underline{E\left[A_{e}^{z}{|}_{\omega_{g}=\omega }\right]=\;A_{z}^{0:\tau }-E\left[\epsilon_{D}^{0:\tau }{\left(M^{0:\tau }\right)}^{T}H^{T}{\left(\Gamma^{\tau }\right)}^{-1}HM^{0:\tau }\epsilon_{D}^{0:\tau }\right]},$$

which shows that in‐breeding (the last term) leads to a consistent underestimation of the real analysis covariance, a result previously found to hold for ensemble Kalman filters (Houtekamer and Mitchell, 1998; van Leeuwen, 1999; Sacher and Bartello, 2005).

## 4 ILLUSTRATION WITH A NUMERICAL EXPERIMENT

In this simple example we illustrate the difference between direct and indirect sampling errors. We use a one‐step assimilation window and estimate initial conditions and one model jump. In this case the temporal correlation errors play no role. No cycling is performed in this experiment.

We let the size of the system be *N*_*x*_ = 250, **H** = **I**, **M** = *m* **I**, **B** = *b* ^2^ **I**, **R** = *r* ^2^ **I**, **Q** = *q* ^2^ **I**. The total covariance becomes **Γ**=*γ* ^2^ **I**, with *γ* ^2^ = *m* ^2^ *b* ^2^ + *q* ^2^ + *r* ^2^. We let all variances be the same *b* = *q* = *r* = 1, and *m* = 1. Let the prior mean of **x** ^0^ be ***μ*** ^0,*b*^ = **0**. For simplicity we use an observation with the same value in all components: **y** = **3**, where $3\in R^{N_{x}}$ is a vector with a 3 in every position. The exact posterior moments are:

(102) $$\begin{array}{ccc} & \mu_{z}^{0:1,a}=\left[\begin{array}{c} \mu_{z}^{0,a}\\ \mu_{\nu }^{1,a} \end{array}\right]=\left[\begin{array}{c} 1\\ 1 \end{array}\right],\; & A_{z}^{0:1}\left[\begin{array}{cc} A_{z}^{0,0} & A_{z}^{0,1}\\ A_{z}^{1,0} & A_{z}^{1,1} \end{array}\right]=\left[\begin{array}{cc} \frac{2}{3}I & -\frac{1}{3}I\\ -\frac{1}{3}I & \frac{2}{3}I \end{array}\right]. \end{array}$$

We generate samples of solutions with different sizes, from *N*_*e*_ = 6 to 6000 members, and we do this in two ways:

- First, we use the real **B** to generate the corresponding gains. In this case all the sampling errors are direct.
- Second, we use the sample estimator **B**_*e*_ for the creation of the gains. In this case, the solutions should both direct and indirect sampling errors.

For each sample size we evaluate the quality of the sample estimators ${\bar{z}}_{z}^{0:1}$ and $A_{z,N_{e}}^{0:1}$ with respect to the analytical values given by Equation 102. We do this in the following way:

- The means. Both expected values $\mu_{z}^{0,a}$ and $\mu_{z}^{1,a}$ should be 1 for each one of the *N*_*x*_ = 250 components of the vector, so for each ensemble we take the 250 components of the sample mean and compute the following percentiles: {10, 25, 50, 75, 90}.
- The covariances. $A_{z}^{0:1}$ is formed of four blocks: $A_{z}^{0,0}$, **A**_*z*_, $A_{z}^{1,1}$ and $A_{z}^{0,1}={\left(A_{z}^{1,0}\right)}^{T}$. Given our settings, the matrices are diagonal and constant in their diagonals. For each ensemble we compute $A_{z,N_{e}}^{0:1}$ and we separate the elements into four groups: the diagonal of $A_{00}^{a}$ (the mean of 250 elements), the diagonal of $A_{\nu \nu }^{a}$ (the mean of 250 elements), the diagonals of $A_{0\nu }^{a}$ and $A_{\nu 0}^{a}$ (the mean of 500 elements) and the rest (the mean of 249,000 elements). For each one of the groups we compute the same 5 percentiles.

**Figure 7:**
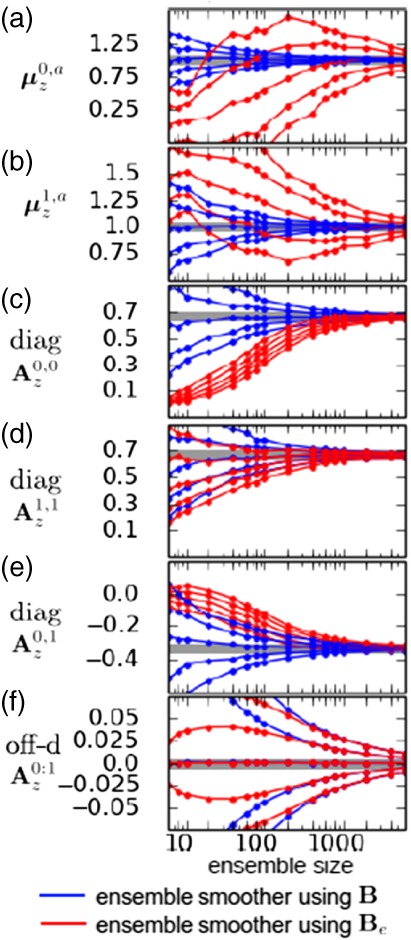
Finite ensemble‐size effects in the weak‐constraint 4D‐Var problem solutions, showing values for the mean (a) at time zero and (b) at the end of the assimilation window, time one. (c)–(f) show the values for different elements of the analysis covariances. The thick grey line indicate the analytical values in each panel. The blue lines represent the percentiles resulting from using **B** in the gains, whereas the red lines represent those resulting from using **B**_e_ in the gains. Note the strong biases in means and diagonal elements in the covariance matrix in the case of an ensemble background covariance [Colour figure can be viewed at wileyonlinelibrary.com]

Figure 7 shows the results of this experiment. Each panel shows a different statistic – (a, b) the means and (c)–(f) the different elements of the covariance matrices. For each panel, the horizontal axis is the ensemble size (in logarithmic scale), and the vertical axis is the value of the estimator. The thick grey line indicates the analytical value in each panel. The blue lines represent the percentiles resulting from using **B** in the gains, and the red lines represent those resulting from using **B**_*e*_ in the gains.

It is clear that the blue estimators only contain direct sampling errors. The spread of the percentiles is symmetric around the expected value, and this spread reduces consistently as the ensemble size increases. The red estimators, on the contrary, present a more complicated behaviour. Looking at the means, there is some bias in the estimation for up to *N*_*e*_≈500, and it has different sign for and $\mu_{z}^{0,a}$ and $\mu_{z}^{1,a}$, and the estimators have large spread. After *N*_*e*_≈500, the spread of the estimator becomes symmetric. This behaviour comes from the nonlinear product $K_{z,\epsilon }^{0:\tau }HM^{0:\tau }{\bar{\zeta }}^{0:\tau ,b}$, which presents a serious bias for small and moderate sample sizes.

In Figure 7c–e, we have the diagonal elements of the analysis covariance blocks. In this case the bias in the estimation of the values is again substantial when using **B**_*e*_. In particular we see small spread but large bias in the estimators (slightly larger for $A_{z}^{1,1}$), which reduces slowly as the sample size grows. This is due to the in‐breeding term $\epsilon_{D}^{0:\tau }{\left(M^{0:\tau }\right)}^{\;T}H^{T}{\left(\Gamma^{\tau }\right)}^{-1}HM^{0:\tau }\epsilon_{D}^{0:\tau }$. The off‐diagonal elements (Figure 7f) show no bias in the estimation, probably because cross‐products of the random matrix elements are uncorrelated.

## 5 SUMMARY AND DISCUSSION

Model errors have been ignored in atmospheric data assimilation far too long. Efforts have been made to correct this in special cases, but it is important to have a basic understanding of the effect of a more general form of model error. In this work we have provided the first systematic exploration of the solution over one time window of the (ensemble) Kalman smoother in the presence of temporal‐correlated model errors, and the consequences of assuming a *guess* correlation time‐scale that is inaccurate.

We have provided exact expressions for the analysis mean and covariance for very general time correlation functions in the model errors. In the univariate case, we performed a deeper exploration of the information flow from observations at the end of the assimilation window to the model variables at different time steps. This information flow is strongly dependent of the magnitude of the model (compressing, identity or expanding) and the magnitude of temporal correlation in the model error.

We have then moved to situations in which our knowledge of the temporal correlations in the model errors in imperfect. First, we considered using a guess for the model error memory, as this is the case often encountered in practice. For instance, fully independent model errors (zero memory) are often represented as fixed by intervals, or fixed in the whole assimilation window (infinite memory). This is done to reduce the computational expense of the problem (Tremolet, 2006). We derived exact expressions for the biases introduced this way, and found that this practical solution can lead to serious errors in the obtained solutions, and an overestimation of the temporal correlations in the model error leads to worse results than an underestimation.

Next we formulated the exact solution for each ensemble member in the case of an finite‐size ensemble. We identified the direct and indirect sources of sampling error. The direct sampling errors arise even when using the exact **B** in the computation of the gain in the problem. The indirect sampling errors come from the effect of using **B**_*e*_ in the computation of the gain. In particular, we find that the ensemble‐based gain is the exact gain left‐ and right‐multiplied by two factors:

$$K_{z,ge}^{0:\tau }=\left(I+\epsilon_{D}^{0:\tau }{\left(D^{0:\tau }\right)}^{-1}\right)K_{z}^{0:\tau }{\left(I+\Gamma_{g\epsilon }^{\tau }{\left(\Gamma^{\tau }\right)}^{-1}\right)}^{-1}.$$

The left factor comes from the error in the joint background‐model error statistics, while the right factor comes from error in the total covariance. For small errors in both the background and the model error specification, we are able to create an approximate expression for the analysis value for each ensemble member. In this expression we identify the exact solution, direct errors, indirect linear errors, and indirect nonlinear errors.

Finally we computed the finite‐size sample analysis mean and sample analysis covariance. We showed that the mis‐specification of the model error memory leads to a wrong analysis covariance, and to the presence of a bias in the analysis sample mean. The sampling errors vanish as the sample size tends to infinity, but this occurs slowly because of a nonlinear product and can lead to a bias in the ensemble mean in small‐to‐moderate sample sizes, which has not been reported before. In the case of the covariance, the mis‐specification of the memory leads to a bias, and the sampling errors do not vanish, instead they tend to a negative offset of the analysis covariance. This is the so‐called in‐breeding which leads to underestimation of covariances. Although some of these results had been established for the EnKF, this is the first time this is explored within a smoother, and it is done while also exploring the interaction with mis‐specified model error temporal correlations.

It is important to remember that the Kalman smoother and its ensemble approximation (EnKS) are sequential algorithms. This is, the solution to the problem includes applying these algorithms serially on subsequent time windows. This paper has analysed the behaviour over one window, as the extension to multiple windows is straightforward (in the linear case). We have assumed that the true model has temporal error correlations, but the observations do not.

It is true that we only consider these correlations inside the time window and ignore temporal correlations over the boundary of two time windows. In principle one could update the trajectories in the previous window when observations in the new window become available. Another approach is to use overlapping windows (e.g. Bocquet and Sakov, 2014, provide a discussion). Nonetheless, the trajectories in the latest window would not be affected, since the starting point – the state of the system given all observations up to the start of the window – does not change. Therefore, improving trajectories in previous windows would not be useful when the emphasis is on forecasting, so the results of this paper are especially important for that case. If the emphasis is on reanalysis then ignoring temporal correlations over window boundaries would become important if the temporal correlations are long compared to the window length.

Our next step will be to move to more realistic systems, for instance in the presence of nonlinear model operators and observational processes. In these cases the effect of cycling is not straightforward, and this will be explored in detail. Our experiments will use a system similar to that of Bonavita *et al.* (2016), but with additive model error instead of multiplicative one (in first instance). Since the solution of the nonlinear problem is a recursion of linearized problems, the results of this paper will provide guidance, but it is clear that many more numerical simulations will be needed in that case.

## ACKNOWLEDGEMENTS

The authors acknowledge the support of the UK National Centre for Earth Observation (NCEO) and PJvL is also supported by the European Research Council (ERC) via the CUNDA project under the European Union's Horizon 2020 research and innovation programme. The useful contributions of three anonymous reviewers are kindly acknowledged.

## APPENDIX

## THE CASE OF L OBSERVATIONAL TIMES IN A TIME WINDOW

In this appendix we generalize Equation 23 – i.e. the expression for the WC Kalman smoother with correlated model errors – to the case when more then one observation time is present in the assimilation window. In this general case we can write the cost function over an assimilation window as

(A1) $$\begin{array}{ll} J\left(z^{0:\tau }\right)= & \frac{1}{2}{\left(z^{0:\tau }-\mu_{z}^{0,b}\right)}^{T}D^{-1}\left(z^{0:\tau }-\mu_{z}^{0,b}\right)\;\\ & +\frac{1}{2}\sum_{l=1}^{L}{\left(y^{l}-Hx^{\theta l}\right)}^{T}R^{-1}\left(y^{l}-Hx^{\theta l}\right).\; \end{array}$$

The sum in the second term corresponds to the *L* observational times. The analysis values of the state variable are obtained as

(A2) $$z^{0:\tau ,a}=\underset{z^{0:\tau }}{arg min}J\left(z^{0:\tau }\right),$$

which in this linear case corresponds to the Kalman equation for the mean. Applying this equation requires writing Equation A1 in a compact form. Let us define the extended observations $y^{1:L}\in R^{LN_{y}}$ as

(A3) $$y^{1:L}={\left[{\left(y^{1}\right)}^{T}\;\text{⋯}\;{\left(y^{L}\right)}^{T}\right]}^{T},$$

the extended observation operator $H^{1:L}\in R^{LN_{y}\times LN_{x}}$ as the rectangular block‐matrix

(A4) $$H^{1:L}=\left[\begin{array}{cccc} H & 0 & \text{⋯} & 0\\ 0 & H & \text{⋯} & 0\\ \vdots & \vdots & \ddots & \vdots \\ 0 & 0 & \text{⋯} & H \end{array}\right],$$

and the extended observation covariance $R^{1:L}\in R^{LN_{y}\times LN_{y}}$ as the block‐diagonal matrix

(A5) $$R^{1:L}=\left[\begin{array}{ccc} R & \text{⋯} & 0\\ \vdots & \ddots & \vdots \\ 0 & \text{⋯} & R \end{array}\right].$$

Now, Equation A1 can be written as

(A6) $$\begin{array}{ll} J\left(z^{0:\tau }\right)= & \frac{1}{2}{\left(z^{0:\tau }-\mu_{z}^{0,b}\right)}^{T}D^{-1}\left(z^{0:\tau }-\mu_{z}^{0,b}\right)\;\\ & +\frac{1}{2}{\left(y^{1:L}-H^{1:L}x^{\theta (1:L)}\right)}^{T}\;\\ & \times {\left(R^{1:L}\right)}^{-1}\left(y^{1:L}-H^{1:L}x^{\theta (1:L)}\right).\; \end{array}$$

All that remains is to express the variables at the times of observations **x** ^*θ*(1:*L*)^ in terms of **z** ^0:*τ*^. This requires applying Equation 14 in each line of the following block vector:

(A7) $$x^{\theta (1:L)}=\left[\begin{array}{c} x^{\theta }\\ x^{2\theta }\\ \vdots \\ x^{L\theta } \end{array}\right]=\left[\begin{array}{c} M^{0:\theta }z^{0:\theta }\\ M^{0:2\theta }z^{0:2\theta }\\ \vdots \\ M^{0:L\theta }z^{0:L\theta } \end{array}\right].$$

We can write Equation A7 in a compact form:

(A8) $$x^{\theta (1:L)}={\tilde{M}}^{0:\theta L}z^{0:\tau }.$$

This can be done if we define the operator ${\tilde{M}}^{0:\theta L}\in R^{LN_{x}\times (\tau +1)N_{x}}$ as a block‐matrix:

(A9) $${\tilde{M}}^{0:\theta L}=\left[\begin{array}{ccccc} M^{0\to \theta } & M^{(1,\theta ):\theta } & 0 & \text{⋯} & 0\\ M^{0\to 2\theta } & M^{(1,\theta ):2\theta } & M^{(\theta +1,2\theta ):2\theta } & \text{⋯} & 0\\ \vdots & \vdots & \vdots & \ddots & \vdots \\ M^{0\to (L-1)\theta } & M^{(1,\theta ):(L-1)\theta } & M^{(\theta +1,2\theta ):(L-1)\theta } & \text{⋯} & 0\\ M^{0\to L\theta } & M^{(1,\theta ):L\theta } & M^{(\theta +1,2\theta ):L\theta } & \text{⋯} & M^{((L-1)\theta +1,L\theta ):L\theta } \end{array}\right],$$

where $M^{((i-1)\theta +1,i\theta ):j\theta }R^{N_{x}\times \theta N_{x}}$ is a modified version of Equation 15. It can be written explicitly as

(A10) $$\begin{array}{ll} & M^{((i-1)\theta +1,i\theta ):j\theta }\;\\ & \;=\left[\begin{array}{c} M^{(i-1)\theta +1\to j\theta }M^{(i-1)\theta +2\to j\theta }\text{⋯}M^{i\theta -1,\to j\theta }M^{i\theta \to j\theta } \end{array}\right].\; \end{array}$$

We are finally ready to write Equation 23 for the case of *L* observational instances in the time window:

(A11) $$\begin{array}{ll} J\left(z^{0:\tau }\right)= & \frac{1}{2}{\left(z^{0:\tau }-\mu_{z}^{0,b}\right)}^{T}D^{-1}\left(z^{0:\tau }-\mu_{z}^{0,b}\right)\;\\ & +\frac{1}{2}{\left(y^{1:L}-H^{1:L}{\tilde{M}}^{0:\theta L}z^{0:\tau }\right)}^{T}{\left(R^{1:L}\right)}^{-1}\;\\ & \times \left(y^{1:L}-H^{1:L}{\tilde{M}}^{0:\theta L}z^{0:\tau }\right).\; \end{array}$$

This equation is solely in terms of **z** ^0:*τ*^. Therefore, all the expressions in sections 2.2 and 2.3 hold if one replaces the elements {**y**,**H**,**M** ^0:*τ*^} with $\left\{y^{1:L},H^{1:L},{\tilde{M}}^{0:\theta L}\right\}$ respectively.
